# Profiles of parents’ emotion socialization within a multinational sample of parents

**DOI:** 10.3389/fpsyg.2023.1161418

**Published:** 2023-08-10

**Authors:** Gabriella L. King, Jacqui A. Macdonald, Christopher J. Greenwood, Christiane Kehoe, Julie C. Dunsmore, Sophie S. Havighurst, George J. Youssef, Tomer S. Berkowitz, Elizabeth M. Westrupp

**Affiliations:** ^1^School of Psychology, Deakin University, Geelong, VIC, Australia; ^2^Centre for Social and Early Emotional Development, Deakin University, Geelong, VIC, Australia; ^3^Department of Pediatrics, The University of Melbourne, Melbourne, VIC, Australia; ^4^Murdoch Children’s Research Institute, Melbourne, VIC, Australia; ^5^Mindful, Department of Psychiatry, The University of Melbourne, Melbourne, VIC, Australia; ^6^Human Development and Family Sciences, Department of Psychological, Health, and Learning Sciences, University of Houston, Houston, TX, United States; ^7^Judith Lumley Centre, La Trobe University, Melbourne, VIC, Australia

**Keywords:** emotion socialization, child emotion development, meta-emotion theory, latent profile analysis, multiple-group latent profile analysis

## Abstract

**Introduction:**

Seminal emotion socialization theories classify parents according to two patterns of parent emotion socialization processes: ‘emotion coaching’ (i.e., parents validate and teach children about emotions) versus ‘emotion dismissing’ parenting (i.e., parents minimize and dismiss their children’s emotions). However, empirical evidence supporting this binary distinction of parents remains limited. Our objective was to investigate whether parents can be differentiated by distinct patterns in their (1) beliefs about children’s emotions, (2) emotion regulation, and (3) emotion-related parenting practices.

**Method:**

Participants were parents of children aged 4–10 years from the Child and Parent Emotion Study (*N* = 869) (https://bmjopen.bmj.com/content/10/10/e038124). Parents completed self-reported measures of emotion socialization processes via an online survey, which took 20–30 min to complete. Data included in the current study were collected May–August 2019. We conducted a latent profile analysis of parents’ emotion socialization (13 indicators). To assess reliability of the profiles, we examined stability of the profiles across (1) parents of children in early versus middle childhood, and (2) fathers versus mothers, via measurement invariance testing. Further, to assess for construct validity of the profiles, we examined concurrent associations between six criterion constructs and parents’ emotion socialization profiles.

**Results:**

A three-profile model emerged characterizing parents by: (1) emotion coaching; (2) emotion dismissing; (3) emotion disengaged. There was strong support for construct validity and reliability.

**Discussion:**

Our study provides empirical support for distinct differentiated classifications of emotion coaching and emotion dismissing parenting, aligned with emotion socialization theories. We further extend on extant theory and suggest a third ‘emotion disengaged’ classification, describing parents with moderate levels of emotion dismissing parenting and low levels of emotion coaching parenting. It should be noted that the profiles were derived with self-report data, therefore, data may have been biased by contextual factors. Furthermore, the study sample consisted of Western families from affluent backgrounds. The field should focus efforts on conducting person-centered studies with more diverse samples in future.

## 1. Background

Emotion competence refers to the ability to manage emotions in an adaptive manner, a skill that is foundational in determining children’s long-term socio-emotional functioning and mental health (Saarni, 1999; Kehoe and Havighurst, 2018). Parents’ emotion socialization is a strong determinant of children’s emotion competence (Morris et al., 2007; Zimmer-Gembeck et al., 2021). Several theoretical frameworks of emotion socialization posit that a number of elements underly parents’ emotion socialization, such as parents’ beliefs about emotions, parents’ emotion regulation, and parents’ emotion-related parenting practices (Gottman et al., 1996; Eisenberg et al., 1998; Morris et al., 2007). While the vast majority of emotion socialization research has utilized a variable-centered approach to examine how these elements are interrelated, there has recently been an emergence of person-centered studies. Person-centered analyses can identify subgroups of parents with similar patterns across several variables, and examine how these subgroups function across certain outcomes, as well as child outcomes (Kusurkar et al., 2021). There is a paucity of person-centered analyses that have examined multiple elements of emotion socialization, with large-scale multinational samples of mothers and fathers. Conducting a latent profile analysis of multiple elements of emotion socialization would enhance emotion socialization theory, as it would allow us to empirically validate Gottman et al.’s (1996) emotion coaching and emotion dismissing parenting constructs. The current study aims to (1) identify multivariate profiles of emotion socialization via a latent profile analysis, and (2) examine construct validity and reliability of the profiles.

Emotion socialization can be defined as the process of children learning culturally relevant beliefs and behaviors related to emotions and emotion expression via everyday social interactions within their environment (Bugental and Goodnow, 1998; Eisenberg et al., 1998; Morris et al., 2007; Friedlmeier et al., 2011). The field of emotion socialization typically posits that several elements of parent emotion socialization influence whether parents are considered emotion coaching or emotion dismissing parents, including: (1) beliefs about emotions, i.e., beliefs parents endorse that are related to emotions and emotion competence, such as the belief that emotions are helpful and important versus the belief that emotions are unhelpful and harmful; (2) parents’ emotion regulation, i.e., the manner in which parents express and regulate their own emotions, often referred to as *implicit* parent emotion socialization; (3) parents’ emotion-related parenting practices, i.e., parenting behaviors that teach children about emotions and scaffold children’s emotion regulation skills (Gottman et al., 1996; Meyer et al., 2014; Ford and Gross, 2018; Hajal and Paley, 2020). The vast majority of emotion socialization research has utilized a variable-centered approach, i.e., examined continuous associations across dimensions of these elements.

Broadly, the field of emotion socialization posits that emotion socialization can be classified into *supportive parenting* (i.e., responses that support, guide and teach children how to regulate their emotions); and *unsupportive parenting* (i.e., parenting that invalidates children’s emotions and does not help children regulate their emotions) (Eisenberg et al., 1998). Supportive/unsupportive parenting align with Gottman et al.’s (1996) conceptualizations of *emotion coaching* and *emotion dismissing* parenting. Emotion coaching parenting includes parents’ beliefs that all emotions can be helpful and important; parents exhibiting adaptive emotion regulation skills; and parents’ supportive and sensitive emotion-related parenting practices, whereby parents encourage and validate their children’s emotions (Gottman et al., 1996; Halberstadt and Eaton, 2003; Katz et al., 2012). In contrast to emotion coaching, emotion dismissing parenting describes parents’ beliefs that do not facilitate child emotion development (e.g., the belief that children use emotions to manipulate others); low levels of emotion regulation; and parents’ unsupportive parenting practices, such as invalidation and minimization (Gottman et al., 1997; Katz et al., 2012). Gottman et al. (1996) and other emotion socialization researchers also contend that parents’ emotion distraction is a dimension of emotion dismissing parenting. It is posited that parents’ emotion distraction does not help guide children’s emotion regulation or facilitate emotion awareness and understanding (Eisenberg, 1996; Magai and O’neal, 1997; Denham and Burton, 2003; Halberstadt et al., 2008; Bjørk et al., 2020). There is strong evidence that emotion coaching parenting is associated with positive child development, such as higher levels of emotion competence, academic success, and social competence (Gottman et al., 1996; Buckholdt et al., 2016; Johnson et al., 2017; Bjørk et al., 2020). Furthermore, strong empirical evidence supports that emotion dismissing parenting is associated with negative child development, such as increased levels of externalizing problems and peer problems, and lower levels of emotion competence (Berlin and Cassidy, 2003; Lunkenheimer et al., 2007; Frankel et al., 2012).

It is widely accepted within emotion socialization theory that associations between parents’ beliefs about emotions, parents’ emotion regulation, and parents’ emotion-related parenting practices are reciprocal. However, there is a paucity of empirical work that has examined the manner in which these elements contribute jointly to parents’ emotion socialization. Unclear measurement precision and conceptualization of the elements underlying parents’ emotion socialization has been a common problem for variable-centered studies. Researchers often conceptualize emotion coaching and emotion dismissing parenting as continuous, unitary variables which combine several dimensions. This approach does not allow researchers to examine the complexity of the dimensions underlying emotion coaching and dismissing constructs. Further, this method cannot provide empirical support for the key proposition of emotion socialization theory, that there are two groupings of parents, since it cannot classify parents into subgroups. While variable-centered approaches examine unitary variables and overlook that some constructs do not exist in isolation (Law and Harrington, 2016), latent profile analysis is a person-centered approach which allows identification of subgroups of participants with similar patterns across several variables (Petersen et al., 2019; Kusurkar et al., 2021). Therefore, it is suitable for modeling complex, multidimensional constructs (Bámaca-Colbert and Gayles, 2010).

Cluster analysis, another type of person-centered analysis, albeit less common, assumes that observations with similar scores across the included variables are a member of the same cluster. On the contrary, latent profile analysis assumes that there are latent profiles which account for patterns of scores across observations (Weller et al., 2020). Two sets of parameters are typically estimated in a latent profile analysis: (1) posterior probabilities which reflect the distribution of profiles within the sample of participants; and (2) item-response means/variances which reflect the profile-specific item means/variances (Witherspoon et al., 2019). The modeling of item-response mean scores allows researchers to examine the nuances of multiple continuous dimensions, i.e., compare the different mean levels of dimensions (e.g., intensity/frequency of beliefs, behaviors etc.) across profiles identified by the analysis. Finally, latent profile analysis can shed light on which patterns of emotion socialization are more or less prominent within a population of parents.

Within the past five years, there has been an increase in studies applying a person-centered approach to parent emotion socialization: (1) Wang et al. (2019): 731 Chinese fathers of children aged 10–18 years; (2) Sosa-Hernandez et al. (2020): 870 ethnoracially diverse US parents (mothers = 419; fathers = 451) of children aged 8–12 years; (3) McKee et al. (2021): 229 parents (mothers = 144, fathers = 85) of children aged 3–12 years from the US; (4) Buhler-Wassmann et al. (2021): 248 ethnoracially diverse US mothers with children aged 3–7 years; (5) Trevethan et al. (2021): 322 Indian and Chinese mothers of children aged 10 to 15 years; (6) Zhu and Dunsmore (2022): 204 Chinese two-parent dyads (mothers = 102, fathers = 102) of children aged 5–10 years; (7) Howe and Zimmer-Gembeck (2022): 322 Australian mothers of children aged 6–8 years. The majority of studies have conducted a latent profile analysis, with a small number of known studies utilizing a cluster analysis (Howe and Zimmer-Gembeck, 2022; Zhu and Dunsmore, 2022).

Almost all previous studies extracted at least an emotion coaching profile, and/or emotion dismissing profile, thus provide empirical support for supportive/emotion coaching and unsupportive/emotion dismissing subgroups of parents (Wang et al., 2019; Sosa-Hernandez et al., 2020; Buhler-Wassmann et al., 2021; McKee et al., 2021; Howe and Zimmer-Gembeck, 2022; Zhu and Dunsmore, 2022). Furthermore, all studies extracted at least two profiles that do not align with emotion coaching or emotion dismissing parenting, which suggests that a binary classification may be insufficient to capture the heterogeneous patterns of parents’ emotion socialization (Wang et al., 2019; Sosa-Hernandez et al., 2020; Buhler-Wassmann et al., 2021; McKee et al., 2021; Trevethan et al., 2021; Zhu and Dunsmore, 2022).

The most common profile identified which did not align with either emotion coaching or emotion dismissing parenting described low-to-moderate levels of emotion coaching and emotion dismissing parenting. Researchers have given these profiles names such as ‘disengaged’, ‘diffuse,’ and ‘low involved’ (Sosa-Hernandez et al., 2020; Buhler-Wassmann et al., 2021; Howe and Zimmer-Gembeck, 2022; Zhu and Dunsmore, 2022). The additional classifications of parent emotion socialization identified beyond emotion coaching and emotion dismissing parenting suggests that levels of engagement in emotion socialization may also be a distinguishing factor between parents.

There are a number of limitations of previous person-centered emotion socialization studies that need to be considered. First, the majority of these studies focused solely on one element of parents’ emotion socialization (i.e., emotion-related parenting practices) (Wang et al., 2019; Sosa-Hernandez et al., 2020; McKee et al., 2021; Trevethan et al., 2021; Zhu and Dunsmore, 2022). One known study has included an assessment of parents’ beliefs about emotions, and parents’ emotion expression, although, this study did not include fathers (Buhler-Wassmann et al., 2021). It should be noted that the aforementioned studies also included the distress reactions subscale from the Coping with Children’s Negative Emotions Scale (Fabes et al., 2002), which several researchers argue is an appropriate proxy measure of parents’ emotion regulation, since it assesses parents’ emotion dysregulation in response to children’s negative emotions (Yagmurlu and Altan, 2009; Hajal and Paley, 2020). However, there is evidence that this subscale has poor construct validity (King et al., 2022), thus, a measure/s of parents’ emotion regulation that has stronger psychometric properties should also be included in future latent profile analyses of parents’ emotion socialization.

An additional limitation of previous studies is that they have not tested whether the profiles identified significantly differ from one another. Examination of the 95% confidence intervals for the profile-specific item means provides evidence that the subgroups of parents significantly differ (Weller et al., 2020). If the 95% confidence intervals are not examined, it is possible that the final model was over-fitted (Weller et al., 2020; Sinha et al., 2021). In a latent profile analysis, each time there are parameters added to the model, i.e., an additional profile, goodness-of-fit indices improve; however, over-fitting of the model becomes more likely (Sinha et al., 2021). Profiles identified in over-fit models are less likely to be replicated in other samples and are less generalizable (Sinha et al., 2021). Examining the 95% confidence intervals can help provide support for profile delineation (Weller et al., 2020; Sinha et al., 2021).

Overall, fathers have been underrepresented in person-centered emotion socialization studies. Variable-centered research has suggested that mothers have higher levels of emotion coaching parenting, and lower levels of emotion dismissing parenting, compared to fathers (Cassano et al., 2007; Nelson et al., 2009; Wong et al., 2009). Further, McKee et al. (2021) latent profile analysis found that mothers were more likely to be assigned to the emotion coaching profile than the emotion dismissing profile, relative to fathers. It is possible that the underrepresentation of fathers in prior studies influenced the profiles that were extracted. Gottman (2001) proposed that fathers are more likely to respond to children’s negative emotions with problem-solving. However, a combination of high levels of problem-solving and only low-moderate levels of emotion dismissing parenting would be considered a pattern of emotion dismissing parenting (Gottman, 2001).

Finally, no known person-centered study of emotion socialization has validated their profiles across different samples, in order to assess reliability of the profiles, via multi-group invariance testing. Multiple-group invariance testing can assess whether latent profiles are equivalent across different groups simultaneously, utilizing goodness-of-fit parameters (Morin et al., 2016; Spurk et al., 2020). Considering construct validity and reliability when conducting person-centered analyses is considered an important part of the process (Spurk et al., 2020). Testing construct validity and reliability of profiles extracted from a latent profile analysis establishes whether they can be generalized beyond the specific sample they were drawn from (Hicks et al. 2017; Petersen et al. 2019).

Findings from previous person-centered analyses provide empirical support to the conceptualization of emotion socialization in terms of distinct parenting profiles (Wang et al., 2019; Sosa-Hernandez et al., 2020; Buhler-Wassmann et al., 2021; McKee et al., 2021; Trevethan et al., 2021; Zhu and Dunsmore, 2022). However, the majority of studies have examined how only one element of emotion socialization contributes to parents’ profiles of emotion socialization. Investigating several elements of emotion socialization, including, beliefs, emotion regulation, and parenting practices, would clarify how these elements occur together within different parent profiles, thus provide a more precise and holistic understanding of how parents socialize their children’s emotions. Previous studies have underrepresented fathers, and only one known study has included a multinational sample of parents (see Trevethan et al. (2021) latent profile analysis of Chinese and Indian parents). The current study aims to identify multivariate profiles of emotion socialization via a latent profile analysis of parents’ self-reported beliefs about children’s emotions, parents’ emotion regulation, and parents’ emotion-related parenting practices. *We predict that the latent profile analysis will extract one or more profiles that align with emotion coaching and emotion dismissing parenting.*

Our second aim is to examine construct validity and reliability of the profiles. To assess reliability of the profiles, we will examine stability of the profiles across (1) parents of children in early versus middle childhood, and (2) fathers versus mothers, via measurement invariance testing. Emotion socialization is thought to be dynamic in nature, changing according to child developmental periods (van der Pol et al., 2015; Mirabile et al., 2016; McKee et al., 2021). Further, strong research evidence suggests that gender influences parent emotion socialization (Cassano et al., 2007; Nelson et al., 2009; McKee et al., 2021). To test construct validity of the profiles, we will examine associations between the profiles and constructs that are theorized to influence parent emotion socialization, i.e., parent gender, familial/parent socio-economic status, parents’ levels of stress, interparental conflict; and theoretically similar constructs, i.e., the family emotional climate, parenting warmth and irritability (Crnic et al., 2005; Morris et al., 2007; Park and Walton-Moss, 2012; Lee and Brophy-Herb, 2018; Sosa-Hernandez et al., 2020; McKee et al., 2021).

## 2. Methods

### 2.1. Participants and study design

The current study drew on data collected within an age-stratified longitudinal cohort study, the Child and Parent Emotion Study (CAPES) (Westrupp et al., 2020). In the current analysis, data consisted of Time 2 data collected for a pilot cohort of participants recruited in 2018 (*n* = 124), and Time 1 data for a second cohort of participants, recruited in 2019 (*n* = 745). Data were collected May–August 2019 via parent-reported online surveys, which took approximately 15–30 min to complete. CAPES was advertised online to prospective parents (i.e., pregnant), and parents of children aged 0–9 years; living in the following countries: Australia, New Zealand, the United Kingdom, Ireland, the United States, and Canada. Advertisements were posted via two main methods: (1) community organizations, such as libraries, paid and unpaid social media ads; and (2) Prolific, a UK-based participant recruitment platform. In the former method, parents were incentivized 20 x AU$50 gift vouchers as a prize for completing the survey. Parents recruited via Prolific were paid after completing the survey. The research team aimed to recruit a more diverse sample in 2019, by targeting advertisements toward fathers, ethnically diverse families, and families from a lower socio-economic status. They were successful in recruiting a higher number of fathers, migrant parents, and single parents. Data were not collected on parents’ race/ethnicity as definitions of these social constructs vary across different countries. Although the study was advertised to parents of children aged up to 9 years, a small number of parents with children above this age completed the survey at Time 1. Further, a small number of parents residing outside of the countries listed under the inclusion criteria provided response data. A more detailed description of participant recruitment and data collection can be found in the Westrupp et al. (2020) protocol paper. The current study was preregistered. Data analysis code for the study is publicly available (see https://osf.io/xtk49/ for preregistration/data analysis code).

### 2.2. Measures

Demographic Characteristics. Demographic characteristics of parents, their partners, and their eldest child were collected via self-report.

Emotion-related parenting practices. Parent-reported emotion-related parenting practices were measured with six subscales from the short-form Coping with Children’s Negative Emotions Scale (King et al., 2022): (1) punitive reactions, i.e., parents’ punitive and controlling behaviors/threat of punishment (3 items, *α* = 0.78); (2) minimization reactions, i.e., parents’ minimization of emotions and derogative comments (3 items, *α* = 0.80); (3) distress reactions, i.e., parents’ emotion dysregulation and distress (3 items, *α* = 0.78); (4) expressive encouragement, i.e., parents’ encouragement of the experience and expression of emotions (3 items, *α* = 0.85); (5) empathy, i.e., parental empathy of children’s emotions (3 items, *α* = 0.81); (6) problem-solving, i.e., parents’ problem-solving to help manage the situation that led to children’s negative emotions (3 items, *α* = 0.65). In addition, we included three items with high factor loadings from the emotion-focused responses subscale of the Coping with Children’s Negative Emotions Scale (described as ‘emotion distraction’ in the current paper) that were excluded from the short-form Coping with Children’s Negative Emotions Scale: (7) emotion distraction, i.e., i.e., where parents may be warm/comforting, but distract children from their emotions (3 items, *α* = 0.65). Subscales from the short-form Coping with Children’s Negative Emotions Scale and the emotion distraction subscale were assessed on a 7-point Likert scale (1 = *very unlikely*, 4 = *medium,* 7 = *very unlikely*). Subscales were derived as standardized mean scores.

Parent emotion regulation. Parent-reported emotion regulation was measured with a modified version of The Difficulties in Emotion Regulation Scale 16-Item Short-Form (Bjureberg et al., 2016). Three items from the impulse subscale were added to strengthen this element of emotion dysregulation (19 items, *α* = 0.94). Higher scores of the subscale reflect higher levels of emotion dysregulation. Items were assessed on a 5-point Likert scale (1 = *almost never*, 5 = *almost always*). The subscale was derived as a standardized mean score.

Parents’ beliefs about children’s emotions. Parent-reported beliefs about children’s emotions were measured with five subscales from the Parents’ Beliefs about Children’s Emotions Questionnaire (Halberstadt et al., 2013): (1) ‘control’: the belief that children can control emotions by themselves (5 items, *α* = 0.78); (2) ‘autonomy’: the belief children do not need help from others to manage their emotions (7 items, *α* = 0.86); (3) ‘stability’: the belief that children’s emotions are stable (4 items, *α* = 0.71); (4) ‘value of anger’: the belief that children’s experience and expression of anger is helpful (6 items, *α* = 0.77); and ‘manipulation’: the belief that children use emotions to manipulate others (4 items, *α* = 0.84). Items were assessed on a 6-point Likert scale (1 = *strongly disagree*, 6 = *strongly agree*). The anger subscale was reverse coded to aid interpretation of the latent profile analysis. Subscales were derived as standardized mean scores.

Parents’ stress. Parent-reported stress (7 items, *α* = 0.89) was measured with the stress subscale of the Depression Anxiety Stress Scales-21 (Henry and Crawford, 2005). Items were assessed on a 4-point scale (0 = *did not apply to me at all*, 3 = *applied to me very much, or most of the time*). Subscales were derived as standardized mean scores.

Interparental conflict. Parent-reported interparental conflict was measured with two subscales from the Argumentative Relationship Scale, modified from the Co-parental Communication Scale (Australian Institute of Australian Studies, 2005): (1) verbal interparental conflict (4 items, *α* = 0.86); (2) physical conflict (1 item). Items were assessed on a 5-point scale (1 = *never*, 5 = *always*). Subscales were derived as standardized mean scores.

Family emotional climate. Parent-reported family emotional climate was measured with two subscales from the short-form Self-Expressiveness in the Family Questionnaire (Halberstadt et al., 1995): (1) positive emotion expression (12 items, *α* = 0.90); (2) negative emotion expression (12 items, *α* = 0.91). Items were assessed on a 9-point scale (1 = *not at all frequently*, 9 = *very frequently*). Subscales were derived as standardized mean scores.

Parenting warmth and irritability. Parent-reported parenting behaviors were measured with two scales from the Longitudinal Study of Australian Children (Zubrick et al., 2014): (1) parenting warmth (6 items, *α* = 0.89); (2) irritability (5 items, *α* = 0.87). The parenting warmth items were assessed on a 5-point Likert scale (1 = *never/almost never*, 5 = *almost/almost always*), and the parenting irritability items were assessed on a 10-point Likert scale (1 = *not at all*, 10 = *all the time*). Subscales were derived as standardized mean scores.

### 2.3. Data analysis

#### 2.3.1. Latent profile analysis

To address aim one, we conducted a latent profile analysis using Mplus (V 8.6) (Muthén and Muthén, 1998–2021) to identify multivariate profiles of parents’ emotion socialization. We estimated models with 1–10 profiles. Thirteen indicators were included in the latent profile analysis: the standardized mean scores of five subscales for parents’ beliefs about children’s emotions, one total score of parent emotion dysregulation, and seven subscales of emotion-related parenting practices. A small number of participants (*n* = 87) did not provide response data for all three measures included in the latent profile analysis. We conducted Little’s MCAR test via Stata to examine whether their response data were missing completely at random (MCAR). Results suggested that their data were not MCAR (*χ*^2^ [65] =104.03, *p* < 0.01, *N* = 956). We excluded their data, as they may have biased our results. Missing data, i.e., item-level missing data, were handled with Full Information Maximum Likelihood. A robust maximum likelihood estimator (i.e., MLR) was used in all models to account for clustering by household (i.e., where two parents from the same household participated in the study, *n* = 111). Our sample of *N* = 869 meets Nylund-Gibson and Choi (2018) recommendation of ≥300 observations for a latent profile analysis.

To determine the optimal number of profiles, a variety of fit statistics and methods were utilized. For instance, we examined changes in the Akaike, Bayesian, and sample-size adjusted Bayesian values. Accordingly, the model with the lowest values is selected as the best-fitting model, or, when adding a profile does not improve model fit. However, it is common for the Akaike, Bayesian, and sample-size adjusted Bayesian values to decrease with the addition of a profile. Due to this, it is common practice for researchers to also plot the Akaike, Bayesian, and sample-size adjusted Bayesian values on an elbow plot. The model which has the most prominent bend and/or is the point where the lines begin to plateau is considered to be the best fitting model, based on the elbow plot alone (Morin et al., 2016). In addition to the elbow plot, we examined the Vuong-Lo–Mendell–Rubin Likelihood Ratio Test/Lo–Mendell–Rubin Adjusted Likelihood Ratio Test. Values which are statistically significant (*p* < 0.05) indicate improvement of model fit in comparison to a model with one less profile (Morin et al., 2016). We examined the 95% confidence intervals of the within-profile means to assess profile delineation. In addition to fit statistics, deciding on the best-fitting model was guided by qualitative interpretation of the profiles, parsimony, and a sound theoretical rationale (Christensen et al., 2020).

#### 2.3.2. Measurement invariance testing

To address aim two, i.e., reliability of the profiles, we assessed stability of the profiles across parents of children in early childhood versus parents of children in middle childhood, and mothers versus fathers. The current study followed steps 1–4 of Morin et al.’s (2016) multiple-group latent profile analysis steps, in order to test for measurement equivalence of the latent profile analysis for parents of children in early childhood versus parents of children in middle childhood, and mothers versus fathers. The first step establishes configural similarity, i.e., whether the same number of profiles can be extracted across each group. We conducted four latent profile analyses separately for (1) parents of children in early childhood; (2) parents of children in middle childhood; (3) mothers; (4) and fathers. Next, we conducted a multiple-group latent profiles analysis to use as a baseline comparison model. Using Mplus’ ‘knownclass’ function (Muthen and Muthen, 2013), we estimated two baseline models in total, i.e., one for parents of children in early childhood and parents of children in middle childhood, and one for both mothers and fathers. Mean levels of indicators were freely estimated across groups, and variance was freely estimated across groups, but constrained within each group. The second step tests structural similarity, constraining indicator means to be equal across groups, and model fit is compared to the baseline configural model (i.e., two of Akaike, Bayesian, and sample-size adjusted Bayesian values must be lower than the baseline comparison model). The third step involves establishing dispersion similarity, i.e., the variance of profiles is the same across samples (i.e., two of Akaike, Bayesian, and sample-size adjusted Bayesian values lower than the structural similarity model). The variance of profiles is constrained to be equal across groups, in addition to constraining the indicator means to be equal across groups. The fourth step involves establishing distributional similarity, i.e., the sample size of the profiles is the same across groups (i.e., two of Akaike, Bayesian, and sample-size adjusted Bayesian values lower than the dispersion similarity model). The within-group profile probabilities are constrained to be equal across groups, in addition to constraining the variance of profiles and the within-profile means to be equal across groups.

#### 2.3.3. Regression analysis

To address aim two, i.e., construct validity of the profiles, a multinomial logistic regression using Stata (V 15.1) (StataCorp, 2017) was conducted, to test the association between constructs that are theorized to influence parent emotion socialization (i.e., parent gender, socioeconomic status, parents’ stress, interparental conflict), and theoretically related constructs (i.e., the family emotional climate, parenting warmth and irritability) with parents’ emotion socialization profiles. We tested one model, whereby all variables were included. Missing data for the criterion constructs ranged from 2 to 15%. All analyses controlled for clustering of parents using a robust variance estimate, the vce cluster command (Williams, 2000).

## 3. Results

### 3.1. Demographic characteristics

Demographic characteristics of parents, their partners, and one of their children were collected via self-report (Table 1). The final sample consisted of *N* = 869 parents (mothers: *n* = 745, fathers: *n* = 324) of children aged 4–10.6 years (*M* = 7.3, *SD* = 1.9). Of this sample, 111 parent dyads participated (i.e., both parents in the household participated). On average, parents were 37 years old (*SD* = 6.8, range = 20–63 years). Almost half of parents were residents of Australia. Almost half were employed to work full-time hours. The majority of parents reported that they had a partner, and the majority reported that they were the biological parent of their child. Parents were largely from an affluent, middle-upper class background, with more than half of parents reporting a household income of >AU$52,000 and receiving a tertiary degree as their highest level of education. Household income was displayed in different currencies for participants residing outside of Australia (i.e., US$, NZ$, GPB).

**Table 1:** Demographic characteristics of sample.

Characteristic	*N* (%)
Parent sex	
Female	545 (63%)
Male	324 (37%)
Child sex	
Female	430 (49%)
Male	432 (50%)
Non-binary/trans-gender	1 (1%)
Total household income per year before tax ($AU)	
Up to $36,400	183 (21%)
$36,400–$52,000	214 (25%)
$52,000–$90,000	182 (21%)
$90,000–$140,000	100 (12%)
Above $140,000	174 (20%)
Parents’ employment	
Full-time	241 (28%)
Part-time	366 (43%)
Long full-time (>45 h per week)	69 (8%)
Unemployed	180 (21%)
Parents’ highest level of education	
Did not complete high school	14 (2%)
High school	139 (16%)
Trade certificate/diploma/ apprenticeship	192 (22%)
Bachelor degree (with or without honors)	291 (34%)
Postgraduate qualification	221 (26%)
Migration Status	
Parent born outside of country of residence	16%
Parent born in country of residence	84%
Country of residence	
Australia	378 (44%)
New Zealand	19 (2%)
United Kingdom	229 (26%)
Ireland	3 (<1%)
United States of America	198 (23%)
Canada	37 (4%)
Malta	1 (<1%)
Tanzania	1 (<1%)
Chile	1 (<1%)
Germany	1 (<1%)
Greece	1 (<1%)
Relationship status	
Partner	670 (89%)
No partner	85 (11%)
Parents’ relationship with child	
Biological parent	672 (96%)
Adopted parent	5 (1%)
Stepparent	15 (2%)
Foster parent	1 (<1%)
Other type of legal guardian	5 (1%)

Data were missing for several parent and child demographics.

### 3.2. Latent profile analysis

**Figure 1:**
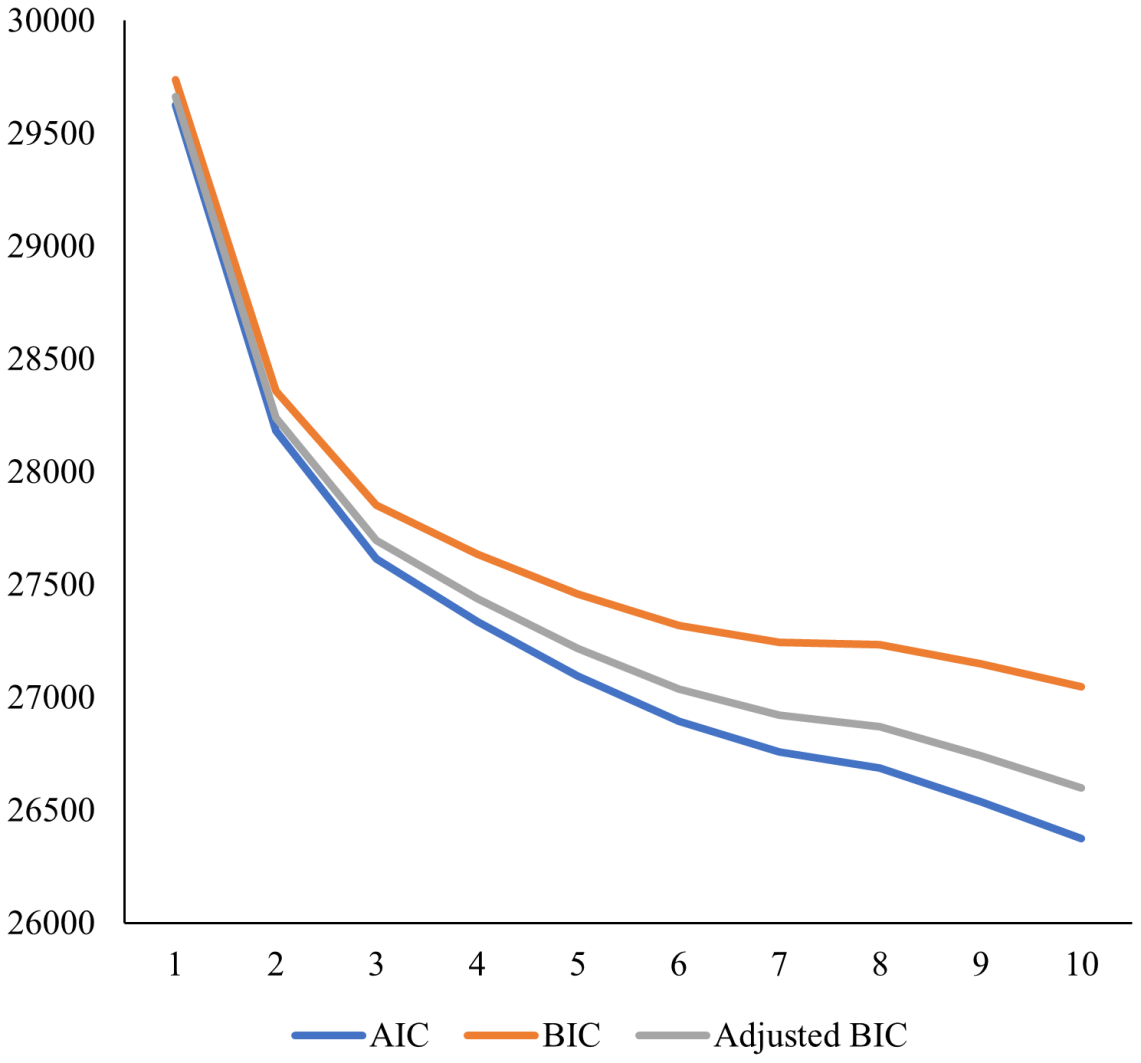
Elbow plot of information criteria for latent profile analysis (*N*  =  869). AIC, Akaike; BIC, Bayesian; SABIC, Sample-Size Adjusted BIC.

**Figure 2:**
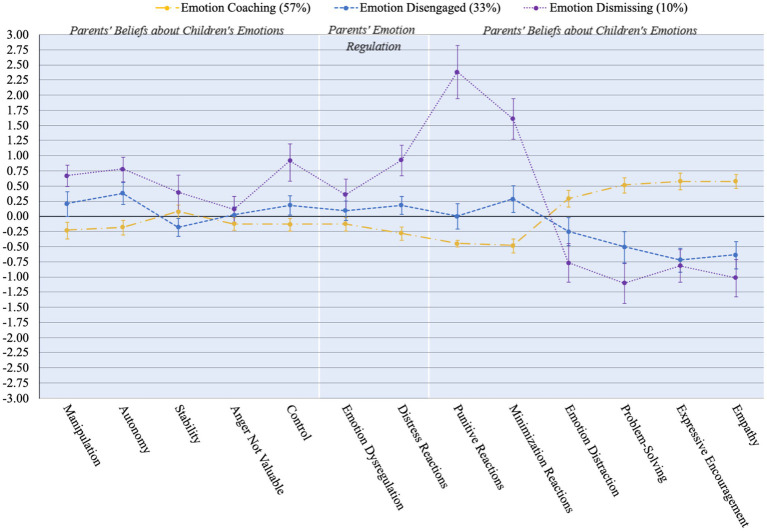
Standardized means scores of the three-profile model indicators and their 95% confidence intervals (*N* = 869).

The three-profile solution was selected as the best-fitting model. Upon inspecting the Akaike (AIC), Bayesian (BIC) and sample-size adjusted Bayesian (ABIC) values plotted on an elbow plot, a bend was visible at the three-profile model, and reductions in the values were smaller for models with additional profiles (see Figure 1). Further, the VLMR and LMR values supported the three-profile model (see Table 2). We plotted and qualitatively examined the standardized mean scores and 95% confidence intervals of the three profiles (see Figure 2). The largest profile (57%, *n* = 492) aligned with ‘emotion coaching’ parenting, whereby parents on average reported low emotion dismissing beliefs (except for moderate levels of the stability belief), low emotion dysregulation, low emotion dismissing parenting practices, and high emotion coaching parenting practices. The smallest profile aligned with ‘emotion dismissing’ parenting (10%, *n* = 86), where parents reported high emotion dismissing beliefs, high emotion dysregulation, very high emotion dismissing parenting practices, and low emotion coaching parenting practices. We describe the third profile (33%, *n* = 291), as ‘emotion disengaged parenting’. On average, parents in this group reported moderate levels of emotion dismissing beliefs, moderate emotion dysregulation, moderate emotion dismissing parenting practices, and low emotion coaching parenting practices. We examined the confidence intervals of the indicators included in the latent profile analysis to assess profile delineation and found that 9 of 13 indicators significantly differed for the emotion coaching versus emotion dismissing profile; 10 of 13 indicators significantly differed for the emotion coaching vs. emotion disengaged profile; and 7 of 10 indicators significantly differed for the emotion dismissing vs. emotion disengaged profile (see Figure 2 and Supplementary Table S2 of the Supplementary materials).

**Table 2:** Goodness-of-Fit statistics for latent profile analyses of parents’ emotion socialization.

Model	*N* FP	LL	AIC	BIC	SABIC	Entropy	VLMR Value of *p*	LMR Value of *p*	*N* (%)
Complete sample									
One	26	−15964.280	31980.560	32104.511	32021.941	-	-	-	869
Two	40	−15189.357	30458.713	30649.407	30522.377	0.92	0.14	0.14	719 (83%) 150 (17%)
Three	54	−14849.385	29806.770	30064.207	29892.716	0.85	0.01	0.01	492 (57%) 291 (33%) 86 (10%)
Four	68	−14677.555	29491.109	29815.289	29599.337	0.88	0.37	0.37	200 (23%) 116 (13%) 497 (57%) 56 (6%)
Parents of children in early childhood									
One	26	−7781.596	15615.191	15720.607	15638.099	-	-	-	426 (100%)
Two	40	−7360.782	14801.564	14963.741	14836.807	0.88	0.24	0.25	309 (73%) 117 (27%)
Three	54	−7174.683	14457.367	14676.306	14504.944	0.87	0.03	0.03	43 (10%) 129 (30%) 254 (60%)
Four	68	−7086.197	14308.394	14584.096	14368.307	0.82	0.03	0.03	121 (28%) 42 (10%) 110 (26%) 153 (36%)
Parents of children in middle childhood									
One	26	−8160.131	16372.262	16478.695	16396.183	-	-	-	427 (100%)
Two	40	−7787.876	15655.752	15819.494	15692.552	0.94	0.24	0.24	374 (84%) 69 (16%)
Three	54	−7632.162	15372.324	15593.377	15422.005	0.84	0.12	0.12	159 (36%) 248 (56%) 36 (8%)
Four	68	−7523.144	15182.288	15460.651	15244.850	0.87	0.15	0.15	127 (29%) 65 (15%) 227 (51%) 24 (5%)
Mothers									
One	26	−9789.479	19630.957	19742.778	19660.244	-	-	-	545
Two	40	−9350.337	18780.674	18952.706	18825.730	0.96	0.12	0.13	58 (11%) 487 (89%)
Three	54	−9140.251	18388.502	18620.745	18449.328	0.86	0.12	0.12	159 (29%) 349 (64%) 37 (7%)
Four	68	−9035.404	18206.807	18499.261	18283.402	0.80	0.43	0.43	165 (30%) 140 (26%) 37 (7%) 203 (37%)
Fathers									
One	26	−5987.140	12026.281	12124.580	12042.111	-	-	-	324
Two	40	−5711.047	11502.095	11653.325	11526.449	0.84	0.34	0.36	309 (73%) 117 (27%)
Three	54	−5589.444	11286.888	11491.048	11319.765	0.84	0.03	0.03	144 (44%) 137 (42%) 43 (13%)
Four	68	−5503.175	11142.350	11399.440	11183.751	0.88	0.29	0.29	4 (1%) 41 (13%) 146 (45%) 133 (41%)

N FP, number of free parameters; LL, loglikelihood; AIC, Akaike; BIC, Bayesian; SABIC, Sample-Size Adjusted BIC; VLMR, Vuon Lo–Mendell–Rubin Likelihood Ratio Test; LMR, Lo–Mendell–Rubin Adjusted Likelihood Ratio Test. See Supplementary Tables S1–S3 for results of the models with 1–10 profiles.

### 3.3. Measurement invariance testing

**Figure 3:**
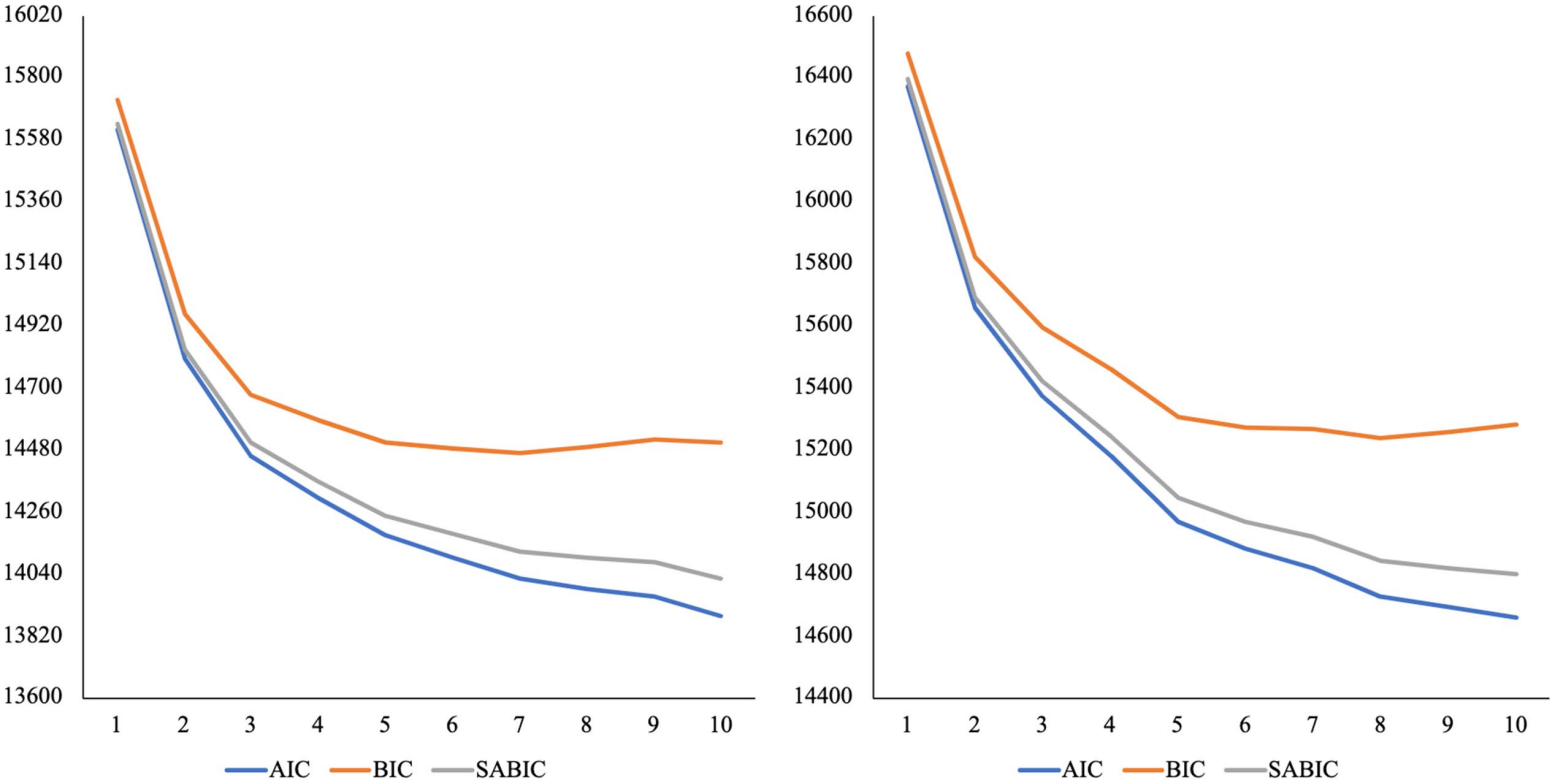
Elbow plots of information criteria for latent profile analysis of parents of children in early childhood (*N* = 426) and middle childhood (*N* = 427). AIC, Akaike; BIC, Bayesian; SABIC, Sample-Size Adjusted BIC.

**Figure 4:**
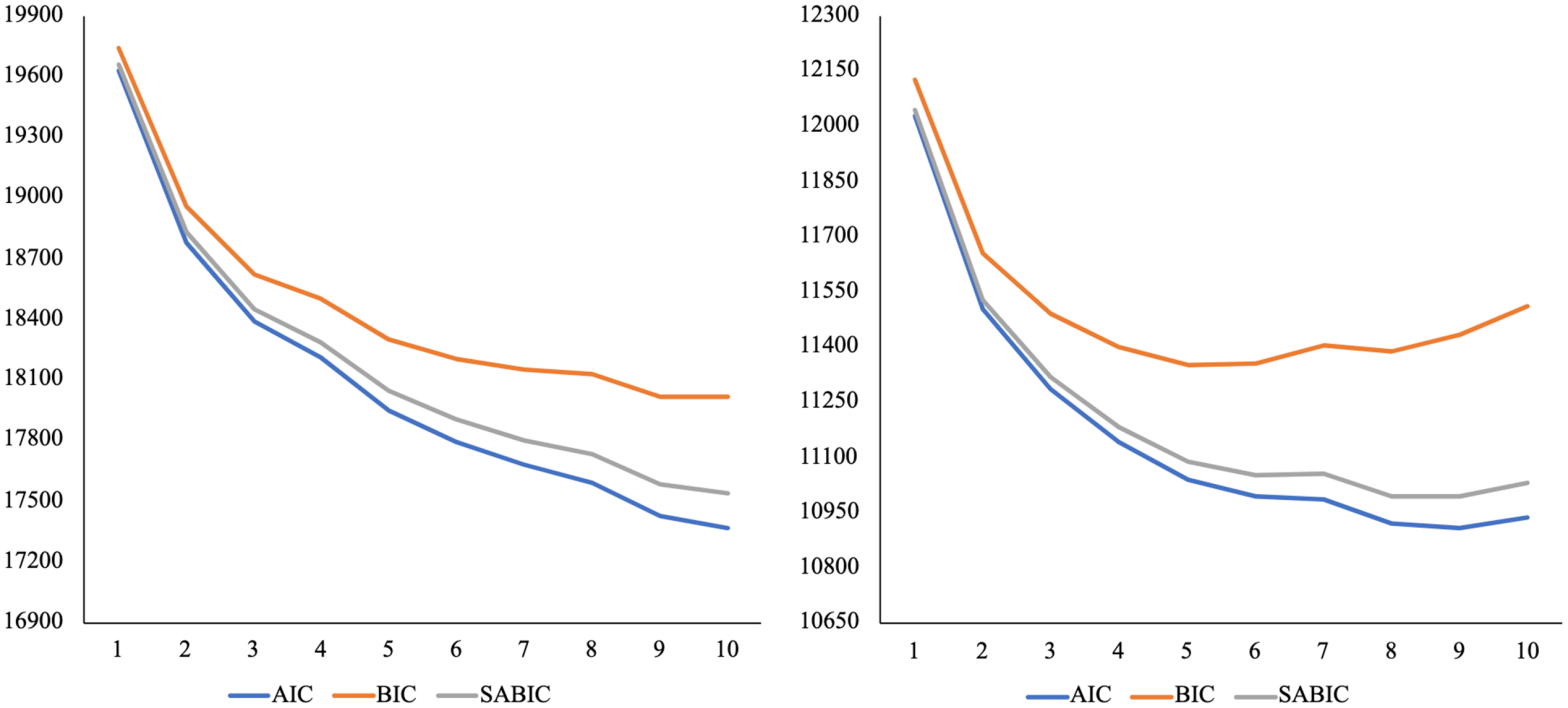
Elbow plot of information criteria for latent profile analysis of mothers (*N* = 545) and fathers (*N* = 324). AIC, Akaike; BIC, Bayesian; SABIC, Sample-Size Adjusted BIC.

We plotted the standardized mean scores of the three-profile models for mothers, fathers, parents of children in early and middle childhood, to examine whether the profiles were similar for each subgroup. It appears that the emotion coaching, emotion disengaged, and emotion dismissing profiles are present for the three-profile solutions (see Supplementary Figures S1–S4). Further, while the AIC, BIC, and ABIC values continue to decline after the three-profile solution for all subgroups, the decline does not reduce after the three-profile model (see Figures 3, 4). The VLMR and LMR *value of p*s were < 0.05 for the three-profile model of fathers and parents of children in early childhood (see Table 2). For mothers and parents of children in middle childhood, *value of p*s were smallest for the six-profile solution. However, the six-profile solutions include profiles with very small numbers of participants. For mothers, there are profiles with solely 3% (*n* = 17) and 1% (*n* = 6) of the sample. For parents of children in middle childhood, there are profiles with solely 3% (*n* = 15) and 4% (*n* = 17) of the sample. While the additional subgroups identified within the six-profile models could be naturally occurring groups, it is difficult to test this. The profiles with <20 parents are likely too small for conducting further tests of construct validity and reliability. Further, some researchers argue that for models with profiles that include <5% of the sample, they may have been overfit (Weller et al., 2020). Conceptually, several of the profiles within the six-profile solutions do not make sense, as they do not align with emotion socialization theory or prior person-centered emotion socialization studies. We continued with the proceeding steps of measurement invariance testing, as we believe there is ample support the emotion coaching, emotion disengaged, and emotion dismissing profiles were identified within all three subgroups.

Two baseline configural models were tested, i.e., the three-profile model was estimated for mothers and fathers, and parents of children in early childhood and parents of children in middle childhood, in multiple-group latent profile analyses. Compared to the baseline configural model, the AIC, BIC, and SABIC values were smaller in the structural model compared to the configural model for parents of children in early childhood versus middle childhood (see Table 3). When comparing the configural model to the structural model for mothers versus fathers, it was found that the AIC value was higher for the structural model, but the BIC and SABIC values were lower for the structural model (see Table 3). Thus, based on fit statistics, there was support for structural similarity across the groups.

**Table 3:** Measurement invariance testing.

Model	k	*N* FP	LL	AIC	BIC	SABIC	Entropy
Parents of children in early childhood versus middle childhood							
Configural similarity	3	107	−15411.404	31036.807	31546.913	31207.107	-
Structural similarity	3	69	−15437.113	31012.225	31341.172	31122.045	0.85
Dispersion similarity	3	56	−15452.300	31016.600	31283.571	31105.729	0.85
Distributional similarity	3	55	−15451.038	31012.077	31274.281	31274.281	0.85
Mothers versus Fathers							
Configural similarity	3	107	−15305.095	30824.190	31334.296	30994.490	0.85
Structural similarity	3	69	−15369.349	30876.698	31205.645	30986.518	0.85
Dispersion similarity	3	56	−15388.980	30889.961	31156.932	30979.090	0.85
Distributional similarity	3	55	−15417.568	30944.568	31206.772	31032.106	0.85

k, number of profiles; N FP, number of free parameters; LL, loglikelihood; AIC, Akaike; BIC, Bayesian; SABIC, Sample-Size Adjusted BIC.

It was found that when comparing the dispersion model to the structural model for children in early versus middle childhood, the AIC value was higher for the dispersion model, but the BIC and SABIC values were lower for the dispersion model (see Table 3). Likewise, for mothers versus fathers, the AIC value was higher for the dispersion model, but the BIC and SABIC values were lower for the dispersion model (see Table 3). These findings provided support for dispersion similarity of the profiles.

We compared the fit statistics for the dispersion and distributional models, and found that for children in early versus middle childhood, the SABIC value was higher for the distributional model, but the AIC and BIC values were lower (see Table 3). Therefore, distributional similarity was supported for children in early versus middle childhood. Our findings provided strong support that the three profiles are equivalent for parents of children in early versus middle childhood, since there was support for configural, structural, dispersion, and distributional similarity. While there was support for configural, structural, and dispersion similarity for mothers versus fathers, we found that distributional similarity was not supported. The AIC, BIC, and SABIC values were all higher for the distributional model compared to the dispersion model, for mothers versus fathers (see Table 3).

According to Morin et al. (2016), if distributional similarity is not supported, there is evidence that one or more of the profiles are more prominent for one group over the other.

Compared to mothers, there was a higher proportion of fathers within the emotion dismissing and emotion disengaged profiles, and a lower proportion of fathers within the emotion coaching profile (emotion coaching profile: 67% mothers, 45% fathers; emotion disengaged profile: 26% mothers, 45% fathers; emotion dismissing profile: 7% mothers, 14% males). Morin et al. (2016) propose that if distributional similarity is not supported, a qualitative inspection is appropriate if there are strong theoretical grounds. We argue that although distributional similarity was not supported, altogether there was sufficient support for measurement invariance of the three-profile model for mothers and fathers.

### 3.4. Associations between familial characteristics and parent emotion socialization profiles

Parent profiles were represented as a categorical variable, i.e., 0 = *emotion coaching* (reference group); 1 = *emotion disengaged*, 2 = *emotion dismissing*. Mplus utilizes posterior probabilities to assign participants into their most likely latent profile, represented by a categorical variable (Petersen et al., 2019); this variable was exported from Mplus into Stata. In a multinomial logistic regression, the profiles were regressed onto theoretically related constructs (see Tables 4, 5). We tested two models, one in which the emotion coaching profile was set as the base category, and another with the emotion disengaged profile as the base category, so that we could compare emotion coaching versus emotion dismissing, emotion coaching versus emotion disengaged, and emotion dismissing versus emotion disengaged.

**Table 4:** Associations between criterion constructs and emotion socialization profiles: emotion coaching as base category.

Criterion construct	Emotion disengaged				Emotion dismissing			
	*RR*	*SE*	UL/LL 95% CI	*p*	*RR*	*SE*	UL/LL 95% CI	*p*
Parent gender	2.3	0.42	1.6, 3.3	**<0.001**	3.1	0.84	1.8, 5.3	**<0.001**
Socioeconomic status								
Parent education	1.1	0.23	0.77, 1.7	0.52	1.6	0.54	0.78, 3.1	0.20
Household income	0.79	0.16	0.53, 1.2	0.25	0.56	0.19	0.30, 1.9	0.08
Parents’ stress	0.90	0.11	0.70, 1.1	0.36	0.77	0.13	0.56, 1.1	0.12
Interparental conflict								
Verbal interparental conflict	0.92	0.11	0.731, 1.2	0.44	0.84	0.14	0.60, 1.2	0.29
Physical interparental conflict	0.93	0.13	0.71, 1.2	0.56	1.1	0.15	0.85, 1.5	0.44
Family emotional climate								
Positive emotions expressed	0.54	0.08	0.41, 0.71	**<0.001**	0.50	0.09	0.35, 0.73	**<0.001**
Negative emotions expressed	1.3	0.16	1.0, 1.7	**0.03**	1.9	0.35	1.3, 2.7	**<0.001**
Parenting behaviors								
Parenting irritability	1.3	0.14	0.71, 1.2	**0.03**	1.7	0.29	1.2, 2.3	**<0.01**
Parenting warmth	0.65	0.08	0.51, 0.83	**<0.001**	0.51	0.08	0.38, 0.69	**<0.001**

RR, relative risk; SE, standard error; CI, upper limit and lower limit confidence intervals. The emotion coaching profile, being a cis mother, parents’ higher education, (i.e., completion of an undergraduate or postgraduate university degree; education was recoded into a binary variable), and a medium/high income (household income was recoded into a binary variable) were used as the base outcomes for categorical variables. *p*-values of significant associations (*p*<0.05) are in bold.

**Table 5:** Associations between criterion constructs and emotion socialization profiles: emotion disengaged as base category.

Criterion construct	Emotion coaching				Emotion dismissing			
	*RR*	*SE*	UL/LL 95% CI	*p*	*RR*	*SE*	UL/LL 95% CI	*p*
Parent gender	0.43	0.08	0.30, 0.62	**<0.001**	1.3	0.35	0.81, 2.3	0.25
Socioeconomic status								
Parent education	0.88	0.18	0.59, 1.2	0.52	1.4	0.47	0.70, 2.7	0.36
Household income	1.3	0.25	0.85, 1.9	0.25	0.71	0.22	0.39, 1.3	0.26
Parents’ stress	1.1	0.14	0.88, 1.4	0.36	0.87	0.13	0.65, 1.2	0.32
Interparental conflict								
Verbal interparental conflict	1.1	0.12	0.87, 1.4	0.44	0.91	0.14	0.67, 1.2	0.54
Physical interparental conflict	1.2	0.15	0.83, 1.4	0.56	1.2	0.11	1.0, 1.4	**0.04**
Family emotional climate								
Positive emotions expressed	1.9	0.26	1.4, 2.4	**<0.001**	0.94	0.13	0.72, 1.2	0.64
Negative emotions expressed	0.77	0.10	0.60, 0.98	**0.03**	1.4	0.26	1.0, 2.0	**0.04**
Parenting behaviors								
Parenting irritability	0.79	0.09	0.64, 0.10	**0.03**	1.3	0.21	0.98, 1.8	0.07
Parenting warmth	1.5	0.19	1.2, 2.0	**<0.01**	0.79	0.10	0.62, 1.0	0.05

RR, relative risk; SE, standard error; CI, upper limit and lower limit confidence intervals. The emotion disengaged profile, being a cis mother, parents’ higher education, (i.e., completion of an undergraduate or postgraduate university degree; education was recoded into a binary variable), and a medium/high income (household income was recoded into a binary variable) were used as the base outcomes for categorical variables. *p*-values of significant associations (*p*<0.05) are in bold.

Results provided support that fathers were more likely to be a member of the emotion dismissing profile compared to mothers, relative to the emotion coaching profile. We found evidence that negative emotions expressed within the family environment and parenting irritability were associated with the emotion dismissing profile, relative to the emotion coaching profile. Positive emotions expressed within the family environment and parenting warmth were negatively associated with the emotion dismissing profile, relative to the emotion coaching profile. There was no evidence of associations between membership of the emotion dismissing profile and socio-economic status, parents’ stress, or interparental conflict, relative to emotion coaching. In the model with emotion disengaged set as the reference category, these associations were in the opposite direction.

Findings supported that, relative to the emotion coaching profile, parents in the emotion disengaged profile were more likely to be fathers than mothers. There was evidence that negative emotions expressed within the family environment and parenting irritability were associated with the emotion disengaged profile, relative to the emotion coaching profile. Our findings suggested that positive emotions expressed within the family environment and parenting warmth were negatively associated with the emotion disengaged profile, relative to the emotion coaching profile. There was no evidence of associations between membership of the emotion disengaged profile and socio-economic status, parents’ stress, or interparental conflict, relative to emotion coaching.

Findings provided evidence that physical interparental conflict and negative emotion expression in the family were associated with the emotion dismissing profile, relative to the emotion disengaged profile. There was no evidence of associations between membership of the emotion dismissing profile and parent gender, socio-economic status, positive emotion expression in the family, parents’ stress, verbal interparental conflict, parenting warmth, and parenting irritability, relative to emotion disengaged.

## 4. Discussion

The current study identified multivariate profiles of parents’ emotion socialization. Our study presents empirical evidence challenging the proposition that patterns of emotion socialization converge around only two configurations, represented by emotion coaching and emotion dismissing parenting (Gottman et al., 1996; Eisenberg et al., 1998). In support of a broader classification framework, we identified a three-profile model: (1) emotion coaching; (2) emotion dismissing; and (3) emotion disengaged. We found evidence supporting the three-profile model in both mothers and fathers, parents of children in early childhood (i.e., child aged 4–6 years), and parents of children in middle childhood (i.e., child aged 7–11 years). Furthermore, our results support construct validity of the model given that the profiles were differentially associated with similar constructs and with constructs theorized to influence parent emotion socialization.

### 4.1. Emotion coaching profile

The profile with the highest number of parents was the emotion coaching subgroup (57%, *n* = 492). The extraction of an emotion coaching profile supported our prediction that at least one of the profiles would align with this pattern of parenting. The emotion coaching profile describes a group of parents who endorse beliefs that are supportive of children’s emotion competence; have strong emotion regulation skills; and exhibit supportive, emotion-validating parenting practices. Findings from variable and person-centered research have provided evidence that emotion coaching parenting scaffolds children’s emotion development and teaches them how to effectively manage their emotions (Morris et al., 2007; Katz et al., 2012; Bjørk et al., 2020; McKee et al., 2021). Our study provides further empirical support for Gottman et al.’s (1996) meta-emotion theory construct, emotion coaching, and more broadly, supportive parenting (Gottman et al., 1996; Eisenberg et al., 1998). Our findings extend on previous person-centered studies that have also identified a pattern of emotion coaching parenting (Wang et al., 2019; Sosa-Hernandez et al., 2020; Buhler-Wassmann et al., 2021; McKee et al., 2021; Howe and Zimmer-Gembeck, 2022; Zhu and Dunsmore, 2022).

### 4.2. Emotion dismissing profile

The smallest profile (10%, *n* = 86) described a subgroup of emotion dismissing parents. We predicted that the latent profile analysis would identify one profile aligned with emotion dismissing parenting, thus our findings supported this prediction. Parents within this profile endorse beliefs that do not facilitate child emotion competence; find it difficult to manage their emotions; and tend to invalidate and minimize their children’s negative emotions. Variable and person-centered research has found that emotion dismissing parenting does not teach children how to effectively manage their emotions, and may in fact lead to children experiencing increased distress (Denham et al., 2007; Johnson et al., 2017). Person-centered studies have found that children of parents within a similar profile have higher levels of internalizing problems, externalizing problems, and negative affect, relative to parents of children within an emotion coaching profile (Wang et al., 2019; McKee et al., 2021; Howe and Zimmer-Gembeck, 2022; Zhu and Dunsmore, 2022). Identification of the emotion dismissing profile provides further support for Gottman et al.’s (1996) emotion dismissing parenting construct, and broader emotion socialization theory’s unsupportive parenting.

Our findings support previous studies that have also identified an emotion dismissing parenting profile (Wang et al., 2019; McKee et al., 2021; Howe and Zimmer-Gembeck, 2022; Zhu and Dunsmore, 2022). Likewise to the majority of previous studies, the profile with the smallest number of parents was the emotion dismissing profile (Wang et al., 2019; McKee et al., 2021; Zhu and Dunsmore, 2022).

### 4.3. Emotion disengaged profile

The identified third parent profile described ‘emotion disengaged’ parenting (33%, *n* = 291). While this profile extends on theoretical conceptualizations of emotion socialization, prior person-centered studies have extracted a similar profile (Sosa-Hernandez et al., 2020; Wang et al., 2020; Buhler-Wassmann et al., 2021; McKee et al., 2021; Trevethan et al., 2021; Zhu and Dunsmore, 2022). Parents in the emotion disengaged profile reported moderate levels of emotion dismissing beliefs, emotion dysregulation, and emotion dismissing practices, as well as low levels of emotion coaching practices. We emphasize that in naming this group ‘emotion disengaged’ parenting we refer to parents’ passiveness toward children’s emotions, and low levels of support and guidance for managing children’s emotions, rather than more broadly referring to parental engagement with their child. Similar patterns have been described in the broader parenting literature (Kawabata et al., 2011; Briere et al., 2017; Shen et al., 2020; Lan, 2021). For instance, Baumrind (1991) proposed a ‘disengaged’ parenting style that describes parents low in warmth, sensitivity, and attentiveness. Our findings extend emotion socialization research by showing that a ‘disengaged’ parent group appears to be evident when simultaneously examining multiple constructs of emotion socialization, and appears to be evident consistently during early and middle childhood, and for mothers and fathers.

In contrast to the sizeable and established literature examining child outcomes of emotion coaching and dismissing parenting, there is less known about child outcomes for this parent profile. However, some person-centered studies have found that compared to children of parents with an emotion coaching profile of parenting, children of parents with an emotion disengaged profile had higher levels of internalizing problems externalizing problems, negative affect, and diurnal stress, lower levels of emotion regulation, and prosocial behavior (Wang et al., 2019; Sosa-Hernandez et al., 2020; Buhler-Wassmann et al., 2021; McKee et al., 2021; Zhu and Dunsmore, 2022).

### 4.4. Construct validity and reliability of the profiles

We found evidence supporting adequate reliability and construct validity of the profiles. First, we found that the profiles were equivalent in early and middle childhood, suggesting that parents’ approach to emotion socialization is not dependent on child age. Second, in relation to parent gender, we found overall evidence supporting equivalence, but we did find that fathers were less likely to be a member of the emotion coaching profile; this is in line with findings from previous studies (Cassano et al., 2007; Nelson et al., 2009; Wong et al., 2009; McKee et al., 2021). It is of note that we included parental empathy in our latent profile analysis, a dimension not included in prior person-centered analyses. Parental empathy of children’s emotions is considered a key aspect of emotion coaching and child emotional development (Stern et al., 2015; Meng et al., 2020). Fathers may have been disproportionally represented in the emotion coaching profile as a result of measurement bias. Research has suggested that self-report measures of empathy are influenced by gender expectations and biases, thus do not adequately capture empathy of fathers (Baez et al., 2017; Macdonald et al., 2022).

We found evidence that fathers were more likely to be assigned to the emotion dismissing and disengaged profiles than the emotion coaching profile. These findings have extended on our understanding of emotion socialization and parent gender (i.e., cis-gender parents). Research has suggested that mothers have higher levels of emotion coaching parenting, and lower levels of emotion dismissing parenting, compared to fathers (Cassano et al., 2007; Nelson et al., 2009; Wong et al., 2009). One known latent profile analysis found that mothers were more likely to be assigned to an emotion coaching profile than an emotion dismissing profile, relative to fathers (McKee et al., 2021). Overall, fathers have been underrepresented in previous variable-centered research.

The profiles were associated with several criterion constructs within expected directions. Compared to parents in the emotion coaching profile, parents within the emotion dismissing and disengaged profiles reported higher levels of negative emotions expressed within the family environment and parenting irritability. Furthermore, parents within the emotion dismissing and disengaged profiles reported lower levels of positive emotions expressed within the family environment and parenting warmth. These findings provide support for validity of the emotion socialization profiles (Sharp and Fonagy, 2008; Bariola et al., 2011; Warmuth et al., 2020; Davis-Kean et al., 2021; Chung et al., 2022). We note that effect sizes of the aforementioned associations for the disengaged profile were attenuated compared to those for the emotion dismissing profile. For instance, emotion dismissing parents reported higher levels of negative emotions expressed within the family environment compared to the disengaged parents. In a regression model with emotion disengaged set as the reference category, we found evidence that parents within the emotion dismissing profile had higher levels of physical interparental conflict and negative emotion expression within the family environment, although these were weak associations. These findings provide support for profile delineation of the emotion dismissing and disengaged profiles.

Although the emotion dismissing and disengaged parenting profiles share some elements, there is evidence that they are distinct patterns of parenting. Previous person-centered studies found that children of emotion dismissing parents had lower levels of emotion regulation and prosocial behavior compared to children of emotion disengaged parents (Wang et al., 2019; Zhu and Dunsmore, 2022). It is of note that we found emotion dismissing parents were higher in punitive responses to children’s emotions, compared to disengaged parents. Frequently responding to children’s emotions with harsh, punitive responses teaches children that emotions are uncomfortable and increases children’s distress (Gottman et al., 1997; Denham et al., 2007; Thompson and Meyer, 2007). While disengaged parenting may not provide children ample guidance and support to manage their emotions, disengaged parents’ lack of harsh, punitive responses to children’s emotions may buffer their children from the more severe negative outcomes. It is worth considering that the absence of parenting practices which are optimal for children’s emotion competence, versus the presence of parenting practices found to have a negative impact on children’s emotion competence, likely has distinct developmental impacts on children’s emotion competence (Little et al., 2019).

We note that ‘emotion distraction’ (i.e., parents offer comfort to children in a way that distracts the child from their negative emotions) was high for parents within the emotion coaching profile. Although relatively high on average, this subscale was rated lower by parents compared to the other emotion coaching parenting practices (e.g., parental empathy). This suggests that parents within this profile respond to their children’s emotions with emotion distraction/comforting behaviors at times, but are more likely to respond with the other dimensions of emotion coaching we assessed.

It is of note that not all indicators included in the latent profile analysis differentiated parents as expected. Firstly, parents within the emotion coaching profile had higher levels of the belief that children’s emotions are stable over time, than the disengaged profile, and no difference was found for the stability belief between the emotion dismissing and emotion coaching profiles, which was unexpected. Perhaps parents’ stability belief is related to the intensity of parents’ belief about the value or danger of children’s emotions, albeit in different directions for the subpopulations in the emotion coaching and emotion dismissing profiles. Halberstadt et al. (2013) theorize that parents’ beliefs that emotions remain stable over time is linked to less supportive emotion-related parenting practices, due to pessimism about effecting change in children’s emotions. Our findings might suggest the contrary. Perhaps, for parents in the emotion coaching profile, believing their children’s emotions are stable motivates emotion coaching behaviors so they can help their child experience well-regulated emotions and establish long-lasting emotional skills. For parents in the disengaged profile, believing their children’s emotions are changeable may motivate a *laissez-faire* attitude such that they engage in emotion-related parenting practices simply to manage behavior in the moment, thereby limiting their engagement in supportive emotion-related parenting practices relative to emotion coaching parents and in unsupportive emotion-related parenting practices relative to emotion dismissing parents. However, these ideas regarding the attributions that may connect parents’ emotion-related beliefs and parenting practices need to be tested through additional empirical research.

It is of note that parents’ emotion regulation for the emotion disengaged profile was not significantly differentiated from the other two profiles, based on the 95% confidence intervals. Hajal and Paley (2020) argue that for emotion socialization research, measures which assess ‘parent-specific’ emotion regulation should be prioritized. While Hajal and Paley (2020) posit that the distress reactions subscale of the Coping with Children’s Negative Emotions Scale (Yagmurlu and Altan, 2009; Hajal and Paley, 2020) is a viable assessment of parents’ emotion regulation, there is evidence of poor construct validity for this subscale (King et al., 2022). Nevertheless, we found that the short-form distress reactions subscale differentiated parents’ emotion regulation as expected, which has a more parsimonious set of items compared to the original subscale (King et al., 2022).

Finally, it is important to note that in the majority of previous emotion socialization person-centered studies, the emotion coaching profile has accounted for roughly a third of the sample (Wang et al., 2019; Sosa-Hernandez et al., 2020; McKee et al., 2021; Howe and Zimmer-Gembeck, 2022; Zhu and Dunsmore, 2022). More than half of our sample was assigned to emotion coaching (57%), which suggests our sample was overrepresented by emotion coaching parents. Furthermore, on average, 15% of previous studies’ samples were assigned to the emotion dismissing profile (Wang et al., 2019; McKee et al., 2021; Zhu and Dunsmore, 2022). In comparison, 10% of our sample was assigned to this profile. The measures used to assess parents’ beliefs and emotion-related parenting practices may have led to an overrepresentation of emotion coaching parents. These measures align with emotion socialization theory’s binary classification of emotion socialization, thus are not designed to capture other patterns of parents’ emotion socialization. Furthermore, it is possible that the lack of diversity within our sample explains this discrepancy. Families in the current study were largely from an affluent, middle-upper class background, and while parents did not provide response data on their race/ethnicity, it is likely that they were from an Anglo-Celtic/European background, due to the intersection of race/ethnicity and socio-economic status.

While a dearth of research has examined the interplay of emotion socialization and socio-economic status, evidence suggests that higher levels of social disadvantage is associated with emotion dismissing parenting (Shaffer et al., 2012; McKee et al., 2021). For instance, McKee et al.’s (2021) latent profile analysis found that compared to low-income families, high income families were less likely to be a member of an emotion dismissing profile. Parents with limited resources and support likely experience increased stressors and difficulties managing their emotions, therefore, emotion dismissing parenting (Shaffer et al., 2012; Burke and Dittman, 2022). Furthermore, parents who have a higher education may be more likely to receive opportunities and resources that educate them on more optimal parenting approaches.

The cultural background of parents is salient in determining their children’s emotion competence (Curtis et al., 2020). To-date, emotion socialization research has largely focused on samples from a Western background. Therefore, the social norms surrounding emotions and emotion competence within these samples needs to be considered. For example, within Western countries, children’s emotion expression is often supported, in-general, but in several cultures emotion expression is less valued (Yang et al., 2020; Ip et al., 2021). For instance, in several Asian countries, especially those with a more collectivist culture, in which social harmony is emphasized, children’s emotion expression is often viewed as disruptive and unhelpful, thus less accepted by parents (Yang et al., 2020; Ip et al., 2021).

While a number of previous person-centered emotion socialization studies have included samples from ethnically diverse and non-Western backgrounds (Wang et al., 2019; Sosa-Hernandez et al., 2020; Trevethan et al., 2021), as well as families experiencing high levels of social disadvantage (McKee et al., 2021), the majority of studies have included parents from one, but not both, of these marginalized groups. Families from intersecting marginalized groups face cumulative pressures and stressors, which likely influences familial functioning (Morris et al., 2007; White et al., 2012; Sarno et al., 2021). Examining these families is important for understanding how they can be better supported, and subsequently improve child emotional outcomes. Only two known person-centered studies to-date have included samples which represent ethnically diverse/non-Western families, as well as families experiencing social disadvantage (Buhler-Wassmann et al., 2021; Zhu and Dunsmore, 2022). Interestingly, while Buhler-Wassmann et al.’s (2021) study included a diverse sample with high levels of social disadvantage, a large proportion of parents (44%) were still assigned to an emotion coaching profile. Although, this sample only included mothers. On the contrary, in Zhu and Dunsmore (2022) study, roughly a third of the sample were assigned to an emotion coaching profile, which included fathers. How contextual factors influence parents’ profiles of emotion socialization, especially race/ethnicity and socio-economic status, remains understudied, and warrants future attention.

### 4.5. Strengths

There are several strengths of the current study. Firstly, we conducted the first known latent profile analysis of parents’ beliefs about children’s emotions, parents’ emotion regulation, and parents’ emotion-related parenting practices. A large number of indicators that reflected these elements of emotion socialization were included; we were able to examine how these dimensions of emotion socialization are reflected in a large-scale, multinational sample of parents, in a holistic and concise manner. The emotion socialization profiles were equivalent for parents of children in early childhood, middle childhood, mothers, and fathers, thus are generalizable to these groups of parents.

### 4.6. Limitations

It should be noted that there are several limitations of the current study. First, all measures were self-reported by parents, thus responses may have been biased. Research has provided evidence that how social desirability bias influences families’ self-report data is nuanced. For instance, evidence suggests that providing objective parent-report assessments of parenting is more difficult for parents compared to parent-report assessments of child outcomes (Bennetts et al., 2016). Furthermore, self-report data do not provide a direct assessment of parents’ emotion socialization. Person-centered studies which utilize a range of data collection methods, such as survey data as well as observational data, would provide more accurate assessment of parents’ emotion socialization profiles. Our sample underrepresented LGBTQI+ families, First Nations families, and families with high levels of social disadvantage; more than half of parents in the current study had received a tertiary education, and income was positively skewed. Since parenting is contextually driven, person-centered emotion socialization research that is representative of marginalized groups and families experiencing social disadvantage is needed to gain a better understanding of emotion socialization within these families. Marginalized groups experience unique parenting challenges compared to the majority group/s, e.g., identity issues, discrimination, and cumulative stressors. Although our sample was multinational, parents were from Western, English-speaking countries, and as such, share some commonality of cultural values that could relate to emotions. Parents were not asked to provide self-report data related to their race/ethnicity, which would have provided more insight into diversity of our sample. However, it is likely that families were predominantly from a European/Anglo-Celtic background. While latent profile analysis is a robust procedure based on fit statistics, selection of the final model is also informed by researcher’s qualitative interpretation (Petersen et al., 2019). Thus, replication of profiles can be limited by researcher decisions. Additionally, sample characteristics can limit replication (Van De Schoot et al., 2017; Petersen et al., 2019). Nevertheless, the profiles extracted in our latent profile analysis corresponded largely with similar analyses, further supporting the validity and generalizability of these groupings of parents.

### 4.7. Conclusion

The current study identified three profiles of parent emotion socialization that reflect theoretically meaningful constructs, via a latent profile analysis. We have extended emotion socialization theory by providing rigurous empirical support for Gottman et al.’s (1996) meta-emotion theory parenting constructs, i.e., emotion coaching and emotion dismissing. Our findings provide evidence for a third classification of parenting, ‘emotion disengaged’. This profile describes parents’ passiveness toward children’s emotions, and low levels of support and guidance for managing children’s emotions. Drawing attention to disengaged parenting could be helpful for clinicians and researchers. Current emotion-focused parenting interventions aim to reduce emotion dismissing parenting and increase emotion coaching parenting (Havighurst et al., 2020). Clinicians and parenting interventions may be able to tailor their services for parents with an emotion disengaged pattern of emotion socialization, thus potentially improve treatment efficacy and retention rates of parents, and subsequent child development outcomes. Future research should aim to replicate the emotion socialization profiles with diverse samples, and assess child outcomes of the profiles.

The increasing popularity of utilizing a person-centred approach to assess parents’ emotion socialization is a positive development in the field. However, researchers need to consider several factors when applying this analytic approach, such as theory; the dimensions included; the measures used; the sample of parents included in the study; transparency of the key decisions and steps made when conducing the latent profile analysis and selecting the final model; delineation of the profiles; validation and reliability of the profiles.

## Data availability statement

The datasets presented in this study can be found in online repositories. The names of the repository/repositories and accession number (s) can be found below: doi: 10.26193/82RCP6.

## Ethics statement

The studies involving human participants were reviewed and approved by Deakin University Human Research Ethics Committee (DUHREC). The patients/participants provided their written informed consent to participate in this study.

## Author contributions

EW, JM, and GK: conception of the project. EW, CK, SH, and JD: conception of the theoretical frameworks underpinning the project. GK, EW, GY, and TB: data collection. GK, EW, JM, and CG: data analysis. EW and JM: supervision. GK, EW, CK, SH, JD, JM, and CG: drafting of the manuscript. All authors contributed to the article and approved the submitted version.

## Funding

GK receives a scholarship stipend from Deakin University to support their PhD candidature, which is an Australian Government Research Training Program (RTP) Scholarship. EW received a grant from the Centre for Social and Early Emotional Development, Deakin University.

## Conflict of interest

The authors declare that the research was conducted in the absence of any commercial or financial relationships that could be construed as a potential conflict of interest.

## Publisher’s note

All claims expressed in this article are solely those of the authors and do not necessarily represent those of their affiliated organizations, or those of the publisher, the editors and the reviewers. Any product that may be evaluated in this article, or claim that may be made by its manufacturer, is not guaranteed or endorsed by the publisher.
