# Supplementary material for: Profiles of parents’ emotion socialization within a multinational sample of parents
Source: Front Psychol. 2023 Aug 10;14:1161418. doi: 10.3389/fpsyg.2023.1161418 (PMC10447894; doi:10.3389/fpsyg.2023.1161418)
Supplement: Supplementary file 1 [file Data_Sheet_1.docx]

Table S1. Goodness-of-Fit Statistics for Latent Profile Analysis of Parents’ Emotion Socialization (N = 869).

| Model | k | *N* FP | LL | AIC | BIC | SABIC | Entropy | VLMR  *p*-value | LMR  *p*-value | *N* (%) |
| --- | --- | --- | --- | --- | --- | --- | --- | --- | --- | --- |
| One | 1 | 26 | -15964.280 | 31980.560 | 32104.511 | 32021.941 | - | - | - | 869 |
| Two | 2 | 40 | -15189.357 | 30458.713 | 30649.407 | 30522.377 | .92 | .14 | .14 | 719 (83%) 150 (17%) |
| Three | 3 | 54 | -14849.385 | 29806.770 | 30064.207 | 29892.716 | .85 | .01 | .01 | 492 (57%) 291 (33%) 86 (10%) |
| Four | 4 | 68 | -14677.555 | 29491.109 | 29815.289 | 29599.337 | .88 | .37 | .37 | 200 (23%) 116 (13%) 497 (57%) 56 (6%) |
| Five | 5 | 82 | -14523.950 | 29211.899 | 29602.821 | 29342.409 | .94 | .10 | .10 | 11 (1%) 236 (27%) 456 (54%) 51 (6%) 115 (13%) |
| Six | 6 | 96 | -14391.951 | 28975.903 | 29433.568 | 29128.695 | .84 | .17 | .18 | 11 (1%) 180 (21%) 111 (13%) 205 (24%) 311 (36%) 51 (6%) |
| Seven | 7 | 110 | -14281.048 | 28782.096 | 29306.504 | 28957.171 | .86 | .63 | .63 | 8 (1%) 180 (21%) 47 (5%) 305 (35%) 197 (23%) 110 (13%) 22 (3%) |
| Eight | 8 | 124 | -14191.164 | 28630.328 | 29221.478 | 28827.685 | .86 | .26 | .26 | 44 (5%) 8 (1%) 21 (2%) 177 (20%) 168 (19%) 302 (35%) 44 (5%) 105 (12%) |
| Nine | 9 | 138 | -14138.606 | 28553.213 | 29211.106 | 28772.852 | .83 | .67 | .67 | 8 (1%) 164 (19%) 47 (5%) 196 (23%) 106 (12%) 179 (21%) 46 (5%) 102 (12%) 21 (2%) |
| Ten | 10 | 152 | -14081.853 | 28467.705 | 29192.341 | 27459.627 | .83 | .65 | .65 | 8 (1%) 161 (19%) 47 (5%) 84 (10%) 107 (12%) 159 (18%) 99 (11%) 137 (16%) 46 (5%) 21 (2%) |

*Note:* k = number of profiles, *N* FP = number of free parameters, LL = loglikelihood, AIC = Akaike, BIC = Bayesian, SABIC = Sample-Size Adjusted BIC, VLMR = Vuon Lo-Mendell- Rubin Likelihood Ratio Test, LMR = Lo-Mendell-Rubin Adjusted Likelihood Ratio Test.

Table S2. Goodness-of-Fit Statistics for Latent Profile Analysis of Parents’ Emotion Socialization (Parents of Children in Early Childhood versus Middle Childhood).

| Model | k | *N* FP | LL | AIC | BIC | SABIC | Entropy | VLMR  *p*-value | LMR  *p*-value | *N* (%) |
| --- | --- | --- | --- | --- | --- | --- | --- | --- | --- | --- |
| *Early Childhood* | | | | | | | | | | |
| One | 1 | 26 | -7781.596 | 15615.191 | 15720.607 | 15638.099 | - | - | - | 426 (100%) |
| Two | 2 | 40 | -7360.782 | 14801.564 | 14963.741 | 14836.807 | .88 | .24 | .25 | 309 (73%) 117 (27%) |
| Three | 3 | 54 | -7174.683 | 14457.367 | 14676.306 | 14504.944 | .87 | .03 | .03 | 43 (10%) 129 (30%) 254 (60%) |
| Four | 4 | 68 | -7086.197 | 14308.394 | 14584.096 | 14368.307 | .82 | .03 | .03 | 121 (28%) 42 (10%) 110 (26%) 153 (36%) |
| Five | 5 | 82 | -7005.302 | 14174.605 | 14507.069 | 14246.852 | .84 | .53 | .53 | 115 (27%) 84 (20%) 149 (35%) 44 (10%) 34 (8%) |
| Six | 6 | 96 | -6952.303 | 14096.606 | 14485.832 | 14181.189 | .86 | .55 | .56 | 5 (1%) 34 (8%) 87 (20%) 111 (26%) 44 (10%) 145 (34%) |
| Seven | 7 | 110 | -6900.996 | 14021.992 | 14467.981 | 14118.910 | .87 | .24 | .24 | 4 (1%) 31 (7%) 20 (5%) 80 (19%) 141 (33%) 107 (25%) 43 (10%) |
| Eight | 8 | 124 | -6870.244 | 13988.488 | 14491.238 | 14097.740 | .87 | .46 | .46 | 6 (1%) 21 (5%) 30 (7%) 101 (24%) 85 (20%) 17 (4%) 35 (8%) 131 (31%) |
| Nine | 9 | 138 | -6841.064 | 13958.128 | 14517.641 | 14079.716 | .88 | .76 | .76 | 1 (1%) 102 (24%) 4 (1%) 42 (10%) 28 (7%) 81 (19%) 27 (6%) 135 (32%) 6 (1%) |
| Ten | 10 | 152 | -6792.637 | 13889.273 | 14505.548 | 14023.196 | .88 | .79 | .79 | 16 (4%) 8 (2%) 107 (25%) 60 (14%) 16 (4%) 104 (24%) 25 (6%) 28 (7%) 41 (10%) 21 (5%) |
| *Middle Childhood* | | | | | | | | | | |
| One | 1 | 26 | -8160.131 | 16372.262 | 16478.695 | 16396.183 | - | - | - | 427 (100%) |
| Two | 2 | 40 | -7787.876 | 15655.752 | 15819.494 | 15692.552 | .94 | .24 | .24 | 374 (84%) 69 (16%) |
| Three | 3 | 54 | -7632.162 | 15372.324 | 15593.377 | 15422.005 | .84 | .12 | .12 | 159 (36%) 248 (56%) 36 (8%) |
| Four | 4 | 68 | -7523.144 | 15182.288 | 15460.651 | 15244.850 | .87 | .15 | .15 | 127 (29%) 65 (15%) 227 (51%) 24 (5%) |
| Five | 5 | 82 | -7403.088 | 14970.177 | 15305.849 | 15045.618 | .89 | .29 | .30 | 15 (3%) 215 (49%) 135 (30%) 61 (14%) 17 (4%) |
| Six | 6 | 96 | -7343.936 | 14879.872 | 15272.855 | 14968.194 | .85 | .05 | .05 | 76 (17%) 15 (3%) 17 (3%) 127 (29%) 146 (33%) 62 (14%) |
| Seven | 7 | 110 | -7299.241 | 14818.483 | 15268.775 | 14919.685 | .85 | .73 | .73 | 30 (7%) 15 (3%) 60 (14%) 112 (25%) 146 (33%) 63 (14%) 17 (4%) |
| Eight | 8 | 124 | -7240.078 | 14728.156 | 15235.758 | 14842.238 | .87 | .80 | .80 | 112 (25%) 30 (7%) 5 (1%) 64 (14%) 59 (13%) 146 (33%) 16 (4%) 11 (2%) |
| Nine | 9 | 138 | -7207.372 | 14690.743 | 15255.656 | 14817.706 | .88 | .50 | .51 | 5 (1%) 9 (2%) 29 (7%) 109 (25%) 16 (4%) 59 (13%) 11 (2%) 63 (14%) 142 (32%) |
| Ten | 10 | 152 | -7176.754 | 14657.508 | 15279.731 | 14797.352 | .90 | .78 | .78 | 10 (2%) 15 (3%) 1 (1%) 102 (23%) 28 (6%) 166 (37%) 26 (6%) 44 (10%) 35 (8%) 16 (4%) |

*Note:* k = number of profiles, *N* FP = number of free parameters, LL = loglikelihood, AIC = Akaike, BIC = Bayesian, SABIC = Sample-Size Adjusted BIC, VLMR = Vuon Lo-Mendell- Rubin Likelihood Ratio Test, LMR = Lo-Mendell-Rubin Adjusted Likelihood Ratio Test.

Table S3. Goodness-of-Fit Statistics for Latent Profile Analysis of Parents’ Emotion Socialization (Mothers versus Fathers).

| Model | k | *N* FP | LL | AIC | BIC | SABIC | Entropy | VLMR  *p*-value | LMR  *p*-value | *N* (%) |
| --- | --- | --- | --- | --- | --- | --- | --- | --- | --- | --- |
| *Fathers* | | | | | | | | | | |
| One | 1 | 26 | -5987.140 | 12026.281 | 12124.580 | 12042.111 | - | - | - | 324 |
| Two | 2 | 40 | -5711.047 | 11502.095 | 11653.325 | 11526.449 | .84 | .34 | .36 | 309 (73%) 117 (27%) |
| Three | 3 | 54 | -5589.444 | 11286.888 | 11491.048 | 11319.765 | .84 | .03 | .03 | 43 (10%) 129 (30%) 254 (60%) |
| Four | 4 | 68 | -5503.175 | 11142.350 | 11399.440 | 11183.751 | .88 | .29 | .29 | 4 (1%) 41 (13%) 146 (45%) 133 (41%) |
| Five | 5 | 82 | -5437.539 | 11039.077 | 11349.098 | 11089.003 | .88 | .38 | .39 | 4 (1%) 74 (23%) 141 (44%) 39 (12%) 66 (20%) |
| Six | 6 | 96 | -5400.678 | 10993.356 | 11356.308 | 11051.805 | .90 |  |  | 4 (1%) 78 (24%) 61 (19%) 141 (44%) 26 (8%) 14 (4%) |
| Seven | 7 | 110 | -5383.921 | 10987.841 | 11403.723 | 11054.814 | .91 | .83 | .83 | 4 (1%) 79 (24%) 59 (18%) 141 (44%) 26 (8%) 1 (1%) 14 (4%) |
| Eight | 8 | 124 | -5335.722 | 10919.444 | 11388.256 | 10994.940 | .86 | .77 | .77 | 4 (1%) 80 (25%) 38 (12%) 79 (24%) 53 (16%) 30 (9%) 13 (4%) 27 (8%) |
| Nine | 9 | 138 | -5316.787 | 10909.574 | 11431.317 | 10993.595 | .87 | .79 | .79 | 47 (15) 28 (9%) 47 (15) 28 (9%) 47 (15) 28 (9%) 47 (15) 28 (9%) |
| Ten | 10 | 152 | -5316.851 | 10937.702 | 11512.375 | 11030.247 | .86 | .73 | .73 | 4 (1%) 30 (9%) 8 (2%) 80 (25%) 29 (9%) 62 (19%) 56 (17%) 15 (5%) 38 (12%) 2 (1%) |
| *Mothers* | | | | | | | | | | |
| One | 1 | 26 | -9789.479 | 19630.957 | 19742.778 | 19660.244 | - | - | - | 545 |
| Two | 2 | 40 | -9350.337 | 18780.674 | 18952.706 | 18825.730 | .96 | .12 | .13 | 58 (11%) 487 (89%) |
| Three | 3 | 54 | -9140.251 | 18388.502 | 18620.745 | 18449.328 | .86 | .12 | .12 | 159 (29%) 349 (64%) 37 (7%) |
| Four | 4 | 68 | -9035.404 | 18206.807 | 18499.261 | 18283.402 | .80 | .43 | .43 | 165 (30%) 140 (26%) 37 (7%) 203 (37%) |
| Five | 5 | 82 | -8892.969 | 17949.939 | 18302.603 | 18042.303 | .83 | .06 | .06 | 154 (28%) 128 (23%) 56 (10%) 190 (35%) 17 (3%) |
| Six | 6 | 96 | -8800.449 | 17792.898 | 18205.774 | 17901.032 | .86 | .02 | .02 | 6 (1%) 17 (3%) 138 (25%) 147 (27%) 55 (10%) 182 (33%) |
| Seven | 7 | 110 | -8728.304 | 17676.608 | 18149.695 | 17800.512 | .86 | .28 | .28 | 16 (3%) 32 (6%) 128 (23%) 132 (24%) 180 (33%) 48 (9%) 9 (2%) |
| Eight | 8 | 124 | -8671.476 | 17590.951 | 18124.249 | 17730.624 | .87 | .83 | .83 | 128 (23%) 32 (6%) 132 (24%) 7 (1%) 15 (3%) 175 (32%) 53 (10%) (1%) |
| Nine | 9 | 138 | -8574.318 | 17424.637 | 18018.145 | 17580.079 | .88 | .55 | .56 | 106 (19%) 32 (6%) 132 (24%) 6 (1%) 8 (1%) 162 (30%) 18 (3%) 28 (5%) 53 (10%) |
| Ten | 10 | 152 | -8530.331 | 17364.662 | 18018.381 | 17535.874 | .87 | .66 | .66 | 6 (1%) 25 (5%) 141 (26%) 67 (12%) 11 (2%) 147 (27%) 24 (4%) 57 (10%) 59 (11%) 8 (1%) |

*Note:* k = number of profiles, *N* FP = number of free parameters, LL = loglikelihood, AIC = Akaike, BIC = Bayesian, SABIC = Sample-Size Adjusted BIC, VLMR = Vuon Lo-Mendell- Rubin Likelihood Ratio Test, LMR = Lo-Mendell-Rubin Adjusted Likelihood Ratio Test.

Figure S1. Standardized Means Scores of the Three-Profile Model Indicators (Parents of Children in Early Childhood, *n*=426).


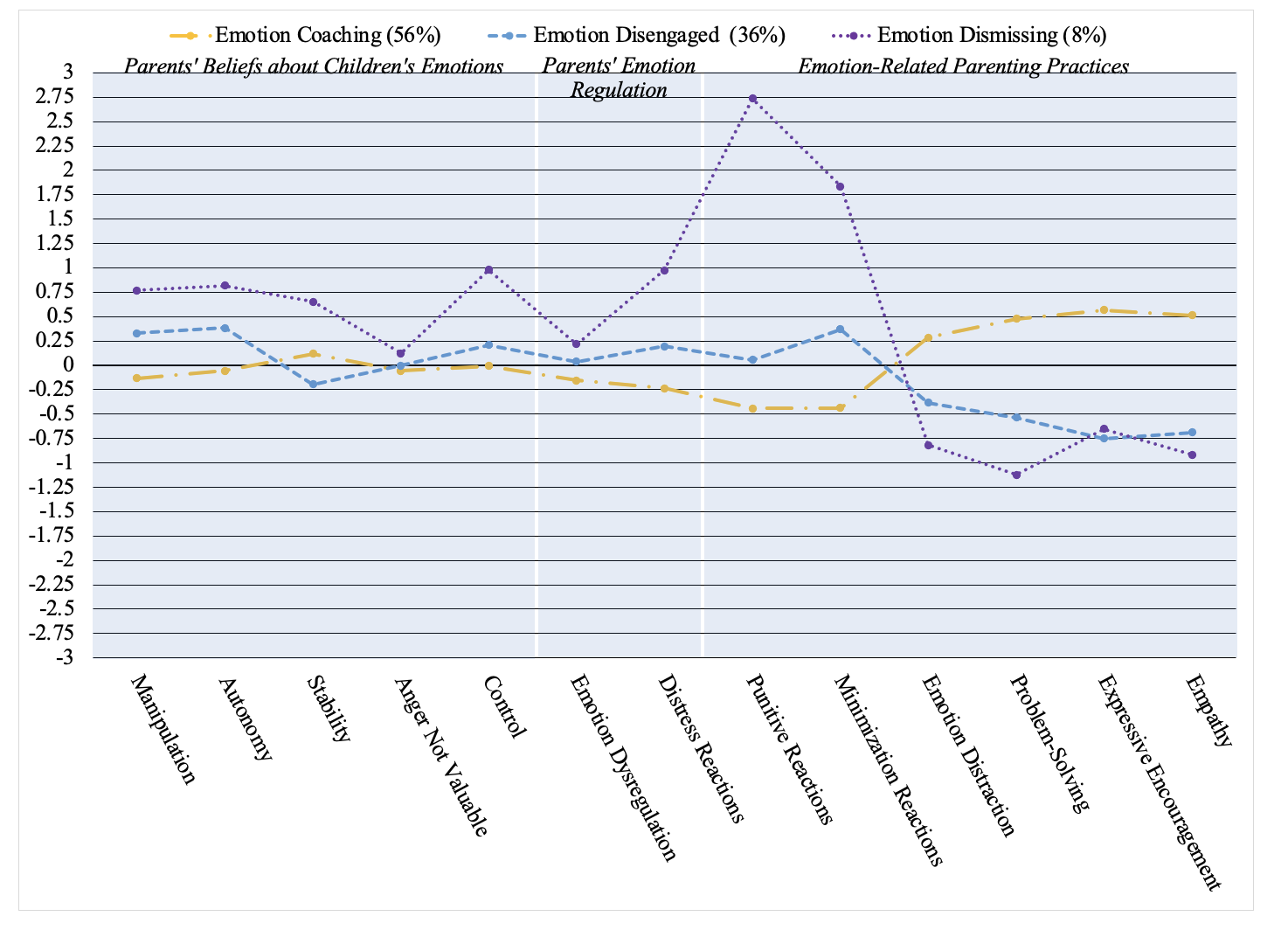


Figure S2. Standardized Means Scores of the Three-Profile Model Indicators (Parents of Children in Middle Childhood, *n*=427).


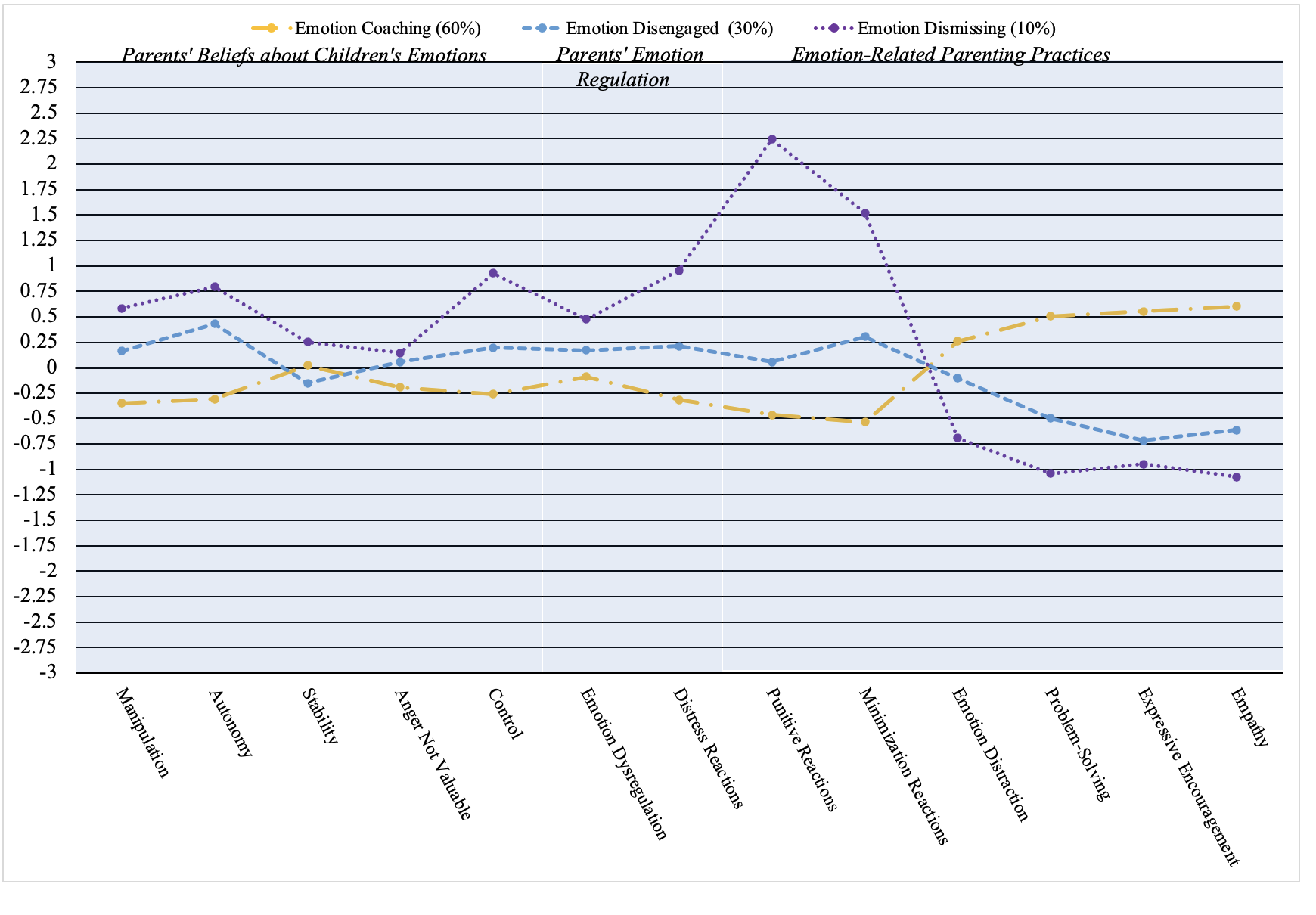


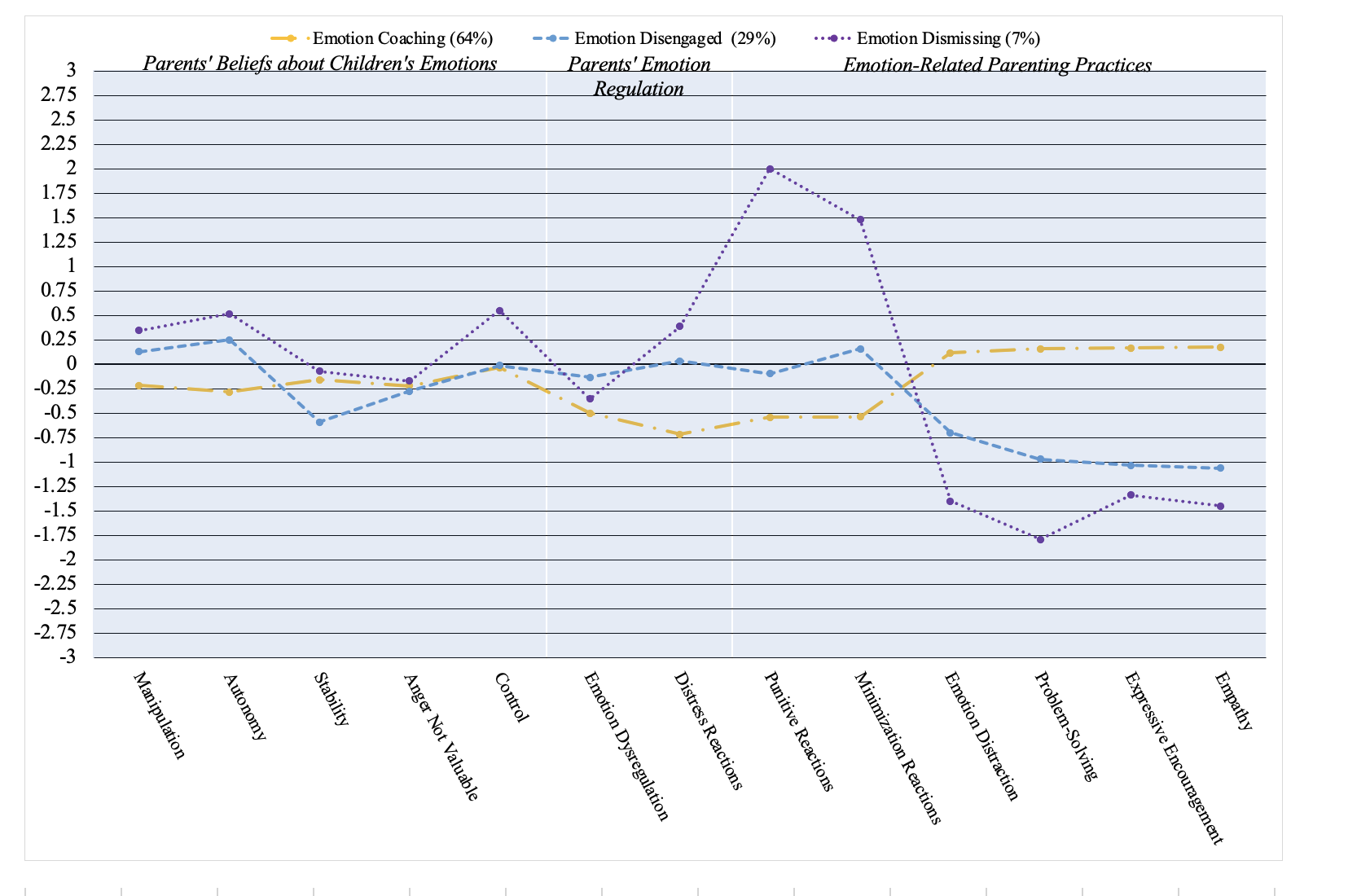
Figure S3. Standardized Means Scores of the Three-Profile Model Indicators (Mothers, *n*=545).

Figure S4. Standardized Means Scores of the Three-Profile Model Indicators for Fathers (*n*=324).


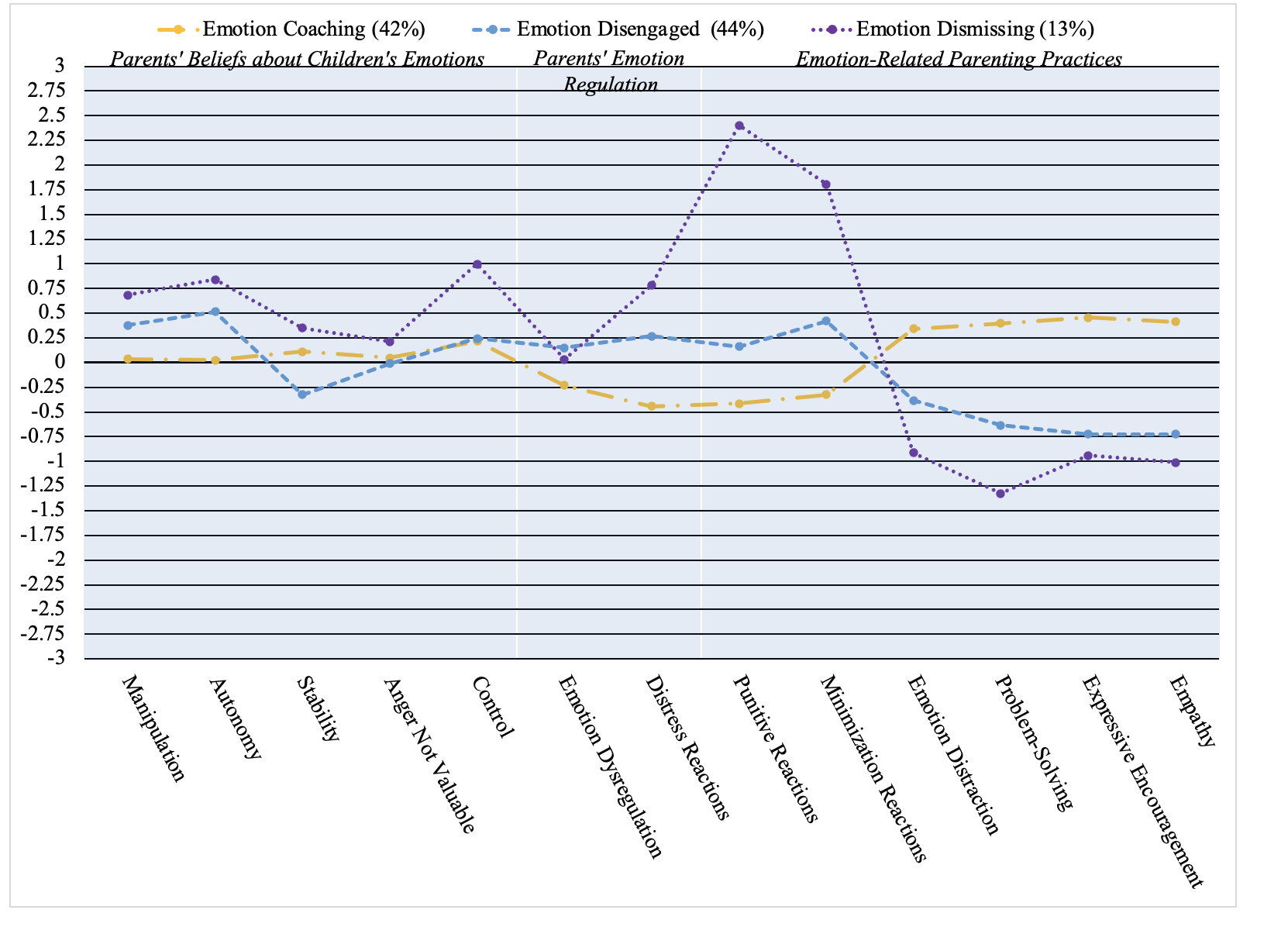


Table S2. Confidence Intervals and Standardized Mean Scores of Within-Profile Indicators (*N*=869).

|  | Emotion Coaching  UL  95% CI | Z-Score | Emotion Coaching  LL  95% CI | Emotion Disengaged  UL  95% CI | Z-Score | Emotion Disengaged  LL  95% CI | Emotion Dismissing  UL  95% CI | Z-Score | Emotion Dismissing  UL  95% CI |
| --- | --- | --- | --- | --- | --- | --- | --- | --- | --- |
| Manipulation | -0.37 | -0.23 | -0.09 | 0.01 | 0.21 | 0.41 | 0.49 | 0.67 | 0.85 |
| Autonomy | -0.30 | -0.18 | -0.07 | 0.21 | 0.38 | 0.56 | 0.58 | 0.78 | 0.98 |
| Stability | -0.03 | 0.08 | 0.19 | -0.33 | -0.18 | -0.03 | 0.11 | 0.40 | 0.68 |
| Anger Not Valuable | -0.23 | -0.12 | -0.02 | -0.11 | 0.02 | 0.15 | -0.09 | 0.12 | 0.33 |
| Control | -0.24 | -0.13 | -0.02 | 0.02 | 0.18 | 0.34 | 0.58 | 0.92 | 1.2 |
| Emotion Dysregulation | -0.24 | -0.13 | -0.02 | -0.07 | 0.10 | 0.26 | 0.10 | 0.36 | 0.61 |
| Distress Reactions | -0.39 | -0.28 | -0.17 | 0.03 | 0.18 | 0.34 | 0.68 | 0.93 | 1.2 |
| Punitive Reactions | -0.50 | -0.45 | -0.39 | -0.20 | 0.00 | 0.21 | 1.9 | 2.4 | 2.8 |
| Minimization Reactions | -0.60 | -0.48 | -0.37 | 0.07 | 0.29 | 0.51 | 1.3 | 1.6 | 1.9 |
| Emotion Distraction | 0.15 | 0.29 | 0.43 | -0.48 | -0.25 | -0.02 | -1.1 | -0.77 | -0.45 |
| Problem-Solving | 0.39 | 0.52 | 0.64 | -0.77 | -0.51 | -0.24 | -1.4 | -1.1 | -0.77 |
| Expressive Encouragement | 0.45 | 0.58 | 0.71 | -0.91 | -0.72 | -0.52 | -1.1 | -0.81 | -0.54 |

*Note.* UL *=* upper limit, LL *=* upper limit, CI = confidence interval.

Table S3. *Posterior Classification Probabilities for Latent Profile Analysis (N*=869).

|  | Emotion Coaching | Emotion Disengaged | Emotion Dismissing |
| --- | --- | --- | --- |
| Emotion Coaching | .95 | .05 | .00 |
| Emotion Disengaged | .09 | .89 | .02 |
| Emotion Dismissing | .00 | .04 | .96 |

*Note.* Posterior classification probabilities ≥ 0.80 provide support for model fit (Spurk, 2020).
